# Fourteen sequence variants that associate with multiple sclerosis discovered by meta-analysis informed by genetic correlations

**DOI:** 10.1038/s41525-017-0027-2

**Published:** 2017-08-08

**Authors:** Sigurgeir Olafsson, Pernilla Stridh, Steffan Daniël Bos, Andres Ingason, Jack Euesden, Patrick Sulem, Gudmar Thorleifsson, Omar Gustafsson, Ari Johannesson, Arni J. Geirsson, Arni V. Thorsson, Bardur Sigurgeirsson, Bjorn Runar Ludviksson, Elias Olafsson, Helga Kristjansdottir, Jon G. Jonasson, Jon Hjaltalin Olafsson, Kjartan B. Orvar, Rafn Benediktsson, Ragnar Bjarnason, Sjofn Kristjansdottir, Thorarinn Gislason, Trausti Valdimarsson, Evgenia Mikaelsdottir, Snaevar Sigurdsson, Stefan Jonsson, Thorunn Rafnar, Dag Aarsland, Srdjan Djurovic, Tormod Fladby, Gun Peggy Knudsen, Elisabeth G. Celius, Kjell-Morten Myhr, Gerdur Grondal, Kristjan Steinsson, Helgi Valdimarsson, Sigurdur Bjornsson, Unnur S. Bjornsdottir, Einar S Bjornsson, Bjorn Nilsson, Ole A. Andreassen, Lars Alfredsson, Jan Hillert, Ingrid Skelton Kockum, Gisli Masson, Unnur Thorsteinsdottir, Daniel F. Gudbjartsson, Hreinn Stefansson, Haukur Hjaltason, Hanne F. Harbo, Tomas Olsson, Ingileif Jonsdottir, Kari Stefansson

**Affiliations:** 1 deCODE genetics/Amgen, Reykjavik, Iceland; 2 Department of Clinical Neuroscience, Karolinska Institutet, Center for Molecular Medicine, Stockholm, Sweden; 3 Institute of Clinical Medicine, University of Oslo, Oslo, Norway; 4 Department of Neurology, Oslo University Hospital, Oslo, Norway; 5 MRC Social, Genetic and Developmental Psychiatry Centre, Institute of Psychiatry, Psychology and Neuroscience, King’s College, London, UK; 6 Integrative Epidemiology Unit, University of Bristol, Bristol, UK; 7 Department of Medicine, Landspitali, the National University Hospital of Iceland, Reykjavik, Iceland; 8 Center for Rheumatology Research, Landspitali, the National University Hospital of Iceland, Reykjavik, Iceland; 9 Children’s Medical Center, Landspitali, the National University Hospital of Iceland, Reykjavik, Iceland; 10 Department of Dermatology, Faculty of Medicine, School of Health Sciences, University of Iceland, Kopavogur, Iceland; 11 Department of Immunology, Landspitali, the National University Hospital of Iceland, Reykjavik, Iceland; 12 Faculty of Medicine, School of Health Sciences, University of Iceland, Reykjavik, Iceland; 13 Department of Neurology, Landspitali, the National University Hospital of Iceland, Reykjavik, Iceland; 14 Department of Pathology, Landspitali, the National University Hospital of Iceland, Reykjavik, Iceland; 15 The Medical Center, Glaesibae, Reykjavik, Iceland; 16 Department of Endocrinology and Metabolic Medicine, Landspitali, the National University Hospital of Iceland, Reykjavik, Iceland; 17 Department of Respiratory Medicine and Sleep, Landspitali, the National University Hospital of Iceland, Reykjavik, Iceland; 18 Department of Medicine, West Iceland Healthcare Centre, Akranes, Iceland; 19 Department of Old Age Psychiatry, Institute of Psychiatry, Psychology, and Neuroscience, King’s College London, London, UK; 20 Division of Psychiatry, Stavanger University Hospital, Stavanger, Norway; 21 Department of Medical Genetics, Oslo University Hospital, Oslo, Norway; 22 Department of Neurology, Akershus University Hospital, Lørenskog, Norway; 23 Norwegian Institute of Public Health, Oslo, Norway; 24 Department of Neurology, Oslo University Hospital Ullevål, Oslo, Norway; 25 Institute of Health and Society, Faculty of Medicine, University of Oslo, Oslo, Norway; 26 Department of Clinical Medicine, University of Bergen, Bergen, Norway; 27 Department of Neurology, Norwegian MS-Registry and Biobank, Haukeland University Hospital, Bergen, Norway; 28 Department of Rheumatology, Landspitali, the National University Hospital of Iceland, Reykjavik, Iceland; 29 Department of Laboratory Medicine, Hematology and Transfusion Medicine, BMC, Lund, Sweden; 30 Division of Mental Health and Addiction, NORMENT, KG Jebsen Centre for Psychosis Research, Oslo University Hospital, Oslo, Norway; 31 Institute of Environmental Medicine, Karolinska Institutet, Stockholm, Sweden; 32 Centre for Occupational and Environmental Medicine, Stockholm County Council, Stockholm, Sweden; 33 School of Engineering and Natural Sciences, University of Iceland, Reykjavik, Iceland

## Abstract

A meta-analysis of publicly available summary statistics on multiple sclerosis combined with three Nordic multiple sclerosis cohorts (21,079 cases, 371,198 controls) revealed seven sequence variants associating with multiple sclerosis, not reported previously. Using polygenic risk scores based on public summary statistics of variants outside the major histocompatibility complex region we quantified genetic overlap between common autoimmune diseases in Icelanders and identified disease clusters characterized by autoantibody presence/absence. As multiple sclerosis-polygenic risk scores captures the risk of primary biliary cirrhosis and vice versa (*P* = 1.6 × 10^−7^, 4.3 × 10^−9^) we used primary biliary cirrhosis as a proxy-phenotype for multiple sclerosis, the idea being that variants conferring risk of primary biliary cirrhosis have a prior probability of conferring risk of multiple sclerosis. We tested 255 variants forming the primary biliary cirrhosis-polygenic risk score and found seven multiple sclerosis-associating variants not correlated with any previously established multiple sclerosis variants. Most of the variants discovered are close to or within immune-related genes. One is a low-frequency missense variant in *TYK2*, another is a missense variant in *MTHFR* that reduces the function of the encoded enzyme affecting methionine metabolism, reported to be dysregulated in multiple sclerosis brain.

## Multiple sclerosis: Sequence variants discovered by meta-analysis informed by genetic correlation

Combining studies and comparing across diseases turned up 14 novel gene variants linked to multiple sclerosis (MS). A team led by Kári Stefánsson and Ingileif Jónsdóttir from deCODE genetics in Reykjavík, Iceland, amalgamated data from a large international study of MS with three smaller ones from Sweden, Norway and Iceland. They conducted a meta-analysis on the combined data set — which encompassed around 21,000 MS patients and 372,000 population controls — and uncovered seven new genetic risk variants linked to MS. The researchers then compared the genetic overlap between various autoimmune diseases in the Icelandic cohort, and documented a close relationship between MS and primary biliary cirrhosis (PBC). They looked more closely at variants linked to PBC, and found that seven also increased the risk for MS, bringing the tally of novel gene variants up to fourteen.

## Introduction

Multiple sclerosis (MS) is a disease in which the oligodendrocytes and the myelin sheets surrounding the axons in the central nervous system are destroyed. Although the causes of MS remain to be determined, it is a widely held opinion that MS is an autoimmune disease (AD) rather than a degenerative neurological disease.^1^ Indeed, genetic studies of MS have yielded more loci influencing immunological than neurological processes and there is a substantial overlap between risk loci for MS and other ADs.^2^

Early genome-wide association studies (GWAS) revealed that many ADs share susceptibility loci.^3^ This motivated the development of the Immunochip, designed to densely cover immune-related loci and loci thought to associate with one or more AD.^4^ Studies using the Immunochip have yielded susceptibility loci for MS,^2^ rheumatoid arthritis (RA),^5^ psoriasis (PSO),^6^ celiac disease (Cel),^7^ type 1 diabetes (T1D),^8^ juvenile idiopathic arthritis (JIA),^9^ primary biliary cirrhosis (PBC),^10^ Crohn’s disease (CD),^11^ ulcerative colitis (UC)^12^ and more. These studies have also revealed considerable overlap of susceptibility loci for many pairs of ADs although the sizes and even the directions of effects vary among diseases.^13^

Polygenic traits, whose risk is affected by a large number of susceptibility variants, require large sample sizes for variants to survive correction for multiple testing in a typical GWAS setting. By establishing prior probabilities, the burden of multiple testing can be reduced. The proxy-phenotype method involves establishing a genetic correlation between two traits and then variants associating with one phenotype serve as “empirically based candidate SNPs” for a second phenotype.^14^ Polygenic risk score (PRS) analyses of many complex traits have shown that a substantial genetic signal resides among the variants not achieving significance in association studies.^15, 16^ While restricting to variants showing a significant association with phenotype A when selecting variants for testing in phenotype B is the approach taken in the proxy-phenotype method,^14^ it is logical to extend this idea to variants that show larger *P*-values of association, provided the additional variants capture genetic effect, as shown with the use of PRSs.

The current study consists of three phases. First, we performed a meta-analyses of publicly available summary statistics from a large study of MS^2^ and three Nordic MS cohorts, identifying seven variants that had not reached genome-wide significance in previously published GWAS. Second, we used PRSs based on public summary statistics (downloaded from Immunobase and IBD Genetics) to quantify the genetic overlap between pairs of ADs in Iceland that is due to common sequence variation, excluding the human leukocyte antigen (HLA) region (due to the extended and complex linkage disequilibrium), finding–among other things–a strong genetic relationship between MS and PBC. Third, utilizing the knowledge of genetic overlap between PBC and MS, we tested the variants contributing to the most predictive PBC-PRS for association with MS, and found seven additional variants affecting risk of MS that have not been previously reported.

## Results

### Meta-analysis

We performed two inverse variance weighted meta-analyses. In the first, which was meant to maximize statistical power for those variants found on the immunochip, we combined publicly available summary statistics from the discovery phase of a large international study of MS, referred to as the IMSGC study^2^ (*N*_case_ = 14,498, *N*_ctrl_ = 24,091), with summary statistics from MS cohorts from Sweden (*N*_case_ = 4505, *N*_ctrl_ = 6105), Norway (*N*_case_ = 1013, *N*_ctrl_ = 23,363) and Iceland (*N*_case_ = 1063, *N*_ctrl_ = 317,639) (Supplementary Table 1). This resulted in combined summary statistics for 117,990 single nucleotide polymorphisms (SNPs) that survived quality control in the IMSGC data and two or more Nordic cohorts. Being from an Immunochip study, the IMSGC data densely covers loci thought to contain candidate genes for ADs, and does not cover the whole genome. The IMSGC cohort further partially overlaps with our Swedish cohort. To search for variants not tagged by any variant on the Immunochip, we performed a second analysis, combining statistics from association testing of imputed genotypes of the three Nordic cohorts, additionally including 1670 cases and 1534 controls in the Swedish cohort that overlapped with the IMSGC study and were therefore excluded from the first analysis. This yielded combined summary statistics for 6,694,339 SNPs that survived quality control in all three studies.

**Fig. 1:**
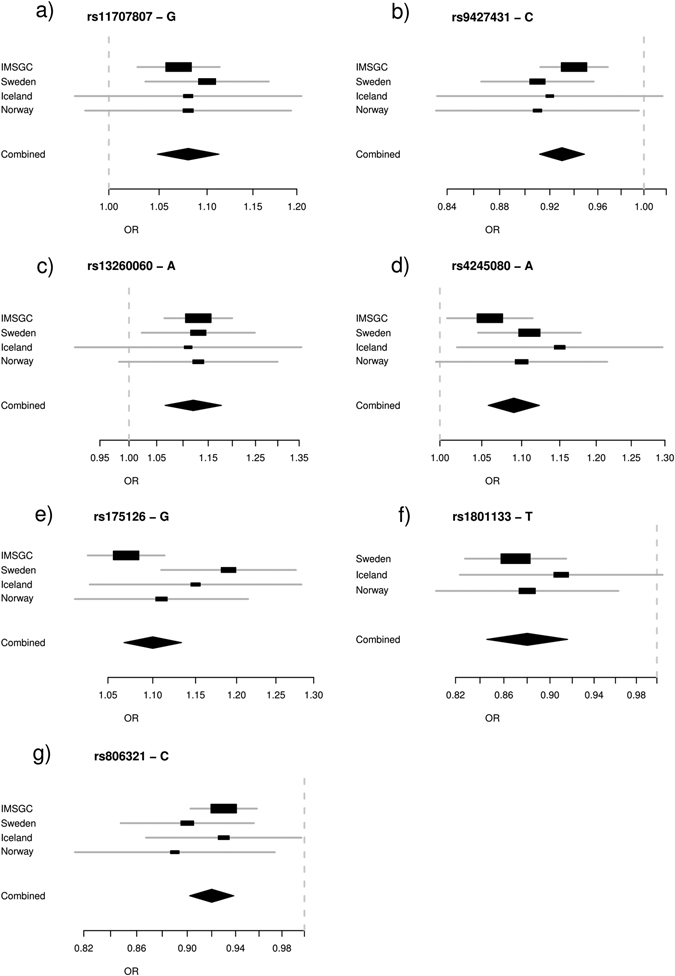
Forest plot for variants discovered in a meta-analysis of MS cohorts. **a** rs11707807; **b** rs9427431; **c** rs13260060; **d** rs4245080; **e** rs175126; **f** rs1801133; **g** rs806321

Excluding the major histocompatibility complex (MHC) region, a total of 94 independent variants reached genome-wide significance with *P* < 5 × 10^−8^ in the first analysis and 24 in the second analysis. Of the newly discovered variants, seven represent signals not previously reported for MS at genome-wide significance level. Six represented loci not previously associated with the disease in GWAS (Table 1, Fig. 1, Supplementary Table 2 and Supplementary Figs. 1–7) while one is a secondary signal at a previously reported locus.

**Table 1:** Meta-analyses identify seven novel risk variants associating with multiple sclerosis

Chr	Position^a^	rsID	MA	OA	MAF [%]	*P*-value	OR (95% CI)	Closest gene(s)	Annotation
1	11796321	rs1801133^b^	T	C	34.4	6.9 × 10^−9^	0.88 (0.84, 0.92)	*MTHFR*	Missense
1	198808343	rs9427431	C	T	39.4	2.2 × 10^−8^	0.93 (0.91, 0.95)	*MIR181A1HG,MIR181B1,MIR181A1,PTPRC,LINC01222,LINC01221*	Intergenic
3	188370473	rs11707807	G	A	41.5	1.2 × 10^−8^	1.08 (1.05, 1.11)	*LPP*	Intronic
8	70306125	rs13260060	A	G	8.5	2.5 × 10^−8^	1.12 (1.08, 1.17)	*NCOA2*	Intronic
11	61066152	rs175126^c^	G	A	45.5	3.3 × 10^−12^	1.10 (1.07, 1.13)	*CD6, CD5*	Intergenic
11	128551691	rs4245080	A	G	47.2	1.5 × 10^−8^	1.09 (1.06, 1.12)	*ETS1*	Intronic
13	50267187	rs806321	C	T	46.1	5.1 × 10^−10^	0.92 (0.90, 0.94)	*DLEU1,ST13P4,DLEU2,MIR15A,MIR16-1,KCNRG*	Intergenic

OR is the OR of the minor allele

*Chr* chromosome number, *MA* minor allele in Iceland, *OA* other allele, *MAF* minor allele frequency in Iceland, *CI* confidence interval

^a^ Build hg38

^b^ Variant not included in the IMSGC study

^c^ After conditioning on rs34383631, previously reported to associate with MS^2^

Only one of our newly discovered MS variants is absent from the Immunochip. The association of rs1801133 with MS was uncovered in the combined analysis of the three Nordic sample sets and is a missense variant is in exon 5 of the *MTHFR* gene, encoding 5,10-methylenetetrahydrofolate reductase, a key enzyme in the intracellular folate (vitamin B9) metabolism and homeostasis. The T allele causes a change of alanine in position 222 to valine. No other sequence variant has a correlation over 0.56 with this variant in Iceland nor in the European 1000 Genomes data, suggesting that rs1801133 itself is causative. This particular mutation has been studied in the context of cardiovascular disease and is known to disrupt the function of 5,10-methylenetetrahydrofolate reductase so that rs1801133(T;C) heterozygotes have 35% less function and rs1801133(T;T) homozygotes have 70% less function of the enzyme than rs1801133(C;C) homozygotes, resulting in increased homocysteine levels in blood.^17^ The minor allele (A) of another variant in *MTHFR*, rs1801131, causes change of glutamate in position 429 to alanine. rs1801131 has also been associated with decreased function of the enzyme but to a lesser degree than rs1801133 and does not associate with the risk of MS in our material (*P* = 0.46, OR = 0.98).

All other variants identified in this phase of the study are intergenic or intronic and none correlate with coding or splice site variants in the Icelandic material (Supplementary Table 3). While rs9427431 and rs175126 are close to genes encoding proteins with roles to play in T-cell adhesion and activation the remaining four variants lie within or close to genes that function as regulators of transcription. In an attempt to understand the mechanism and assess the consequences of all the sequence variants, we explored their effects on expression by looking them up in the Genotype Tissue Expression (GTEx) project^18^ and in data from a large Icelandic study of expression quantitative trait loci (eQTLs) in whole blood,^19^ only linking the associated variant with changes in expression when it is either itself the variant most strongly associated with expression in the locus, or highly correlated (*r* ^2^ > 0.94 in deCODE data) with that variant. We also determined whether the variants had previously been reported to associate with any AD and if they belonged to potential transcription factor binding sites (TFBS) in the ORegAnno database^20^ or likely enhancer elements as defined by histone modification patterns in the ENCODE project.^21^ Lastly, we explored variants correlated with our reported variant in Iceland to search for possible coding or splice site variants that might not have been included on the Immunochip (Supplementary Table 3). The newly identified variants are listed and further discussed in the supplementary note accompanying the paper.

### PRS analysis

**Fig. 2:**
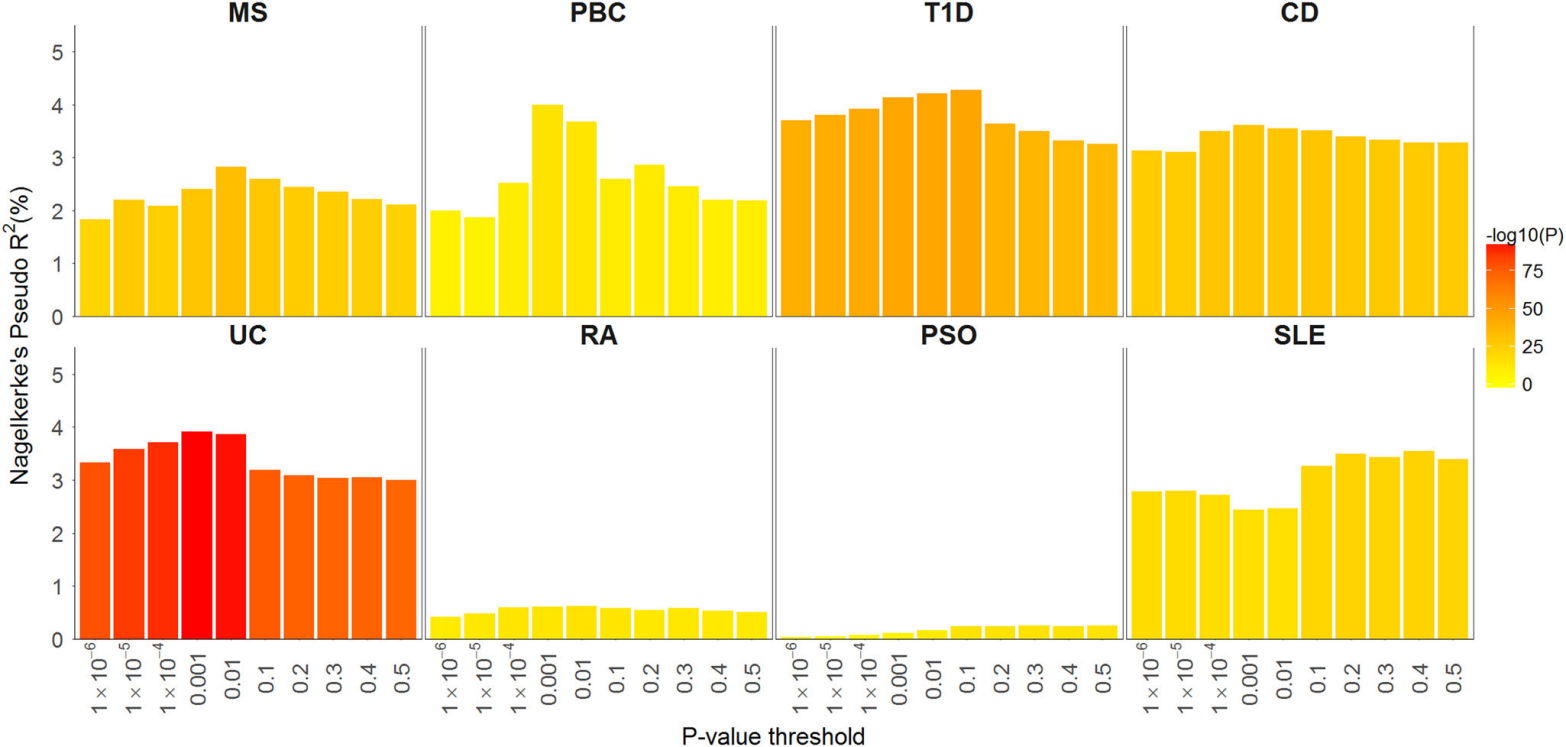
The variance explained by PRS for training diseases predicting the corresponding disease in the Icelandic target cohorts. These bars show how much variance of the phenotype is explained at a given *P*-value threshold by the PRS calculated from the training set for the same phenotype. Celiac disease (Cel) and juvenile idiopathic arthritis (JIA) are not included in the figure as no Icelandic cohorts were available for these diseases. For JIA and Cel, a *P*-value inclusion of 0.001 was arbitrarily selected. *MS* multiple sclerosis; *PBC* primary biliary cirrhosis; *T1D* type 1 diabetes; *CD* Crohn’s disease; *UC* ulcerative colitis; *RA* rheumatoid arthritis; *PSO* psoriasis; *SLE* systemic lupus erythematosus

Risk alleles, *P-*values and effect estimates were extracted from publicly available summary statistics from ten studies of ADs (Supplementary Table 4) and used to define PRSs that were then calculated for 150,656 Icelanders. We excluded the extended MHC region (chr6:25000000–35000000, build hg38) from this analysis because of the number of strong associations with ADs and the complex linkage disequilibrium structure of the region. The association of the AD-PRS was tested with its corresponding disease in Iceland (Supplementary Table 5) at 10 *P-*value inclusion thresholds, and the threshold at which the largest part of the variance in case-control status of Icelanders was explained by the score was identified (Fig. 2 and Supplementary Tables 6 and 7).

**Fig. 3:**
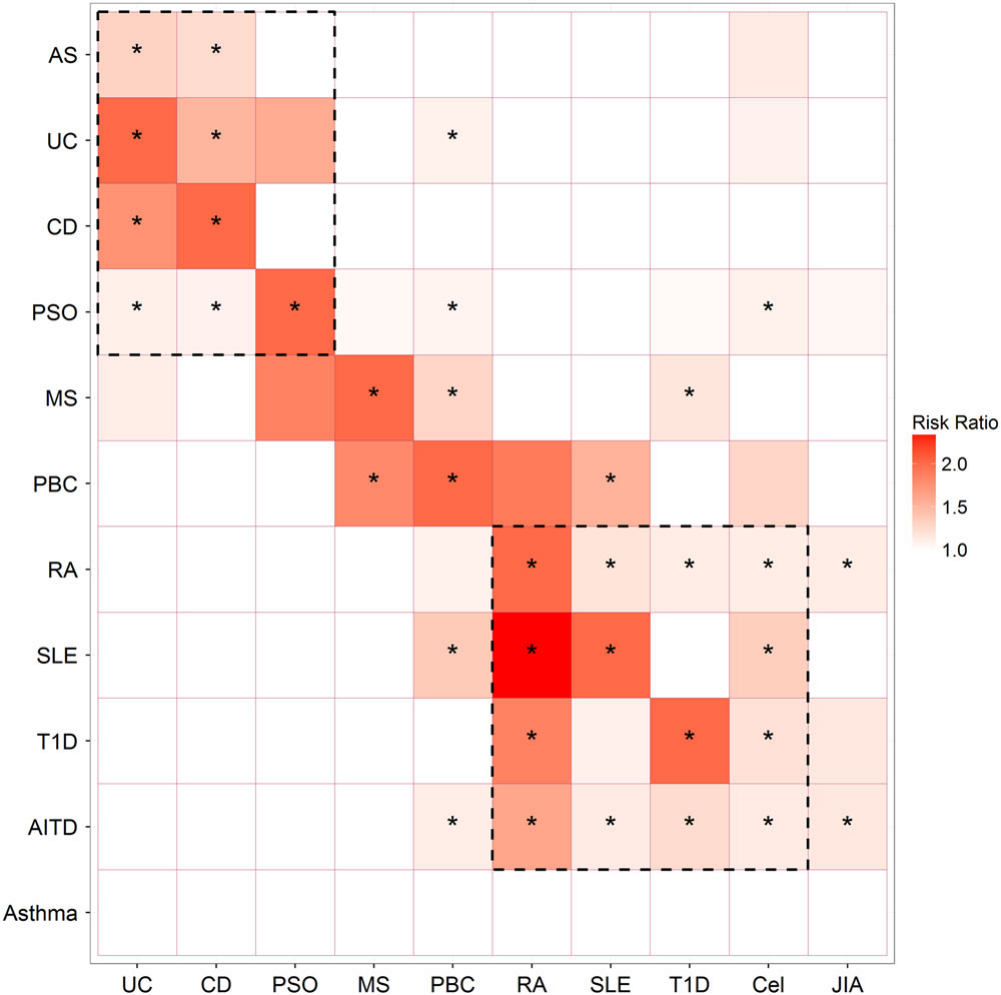
A heat map showing the genetic relationships between autoimmune diseases and asthma. *Squares* labeled with an *asterisk* are significant after correcting for 102 tests. The risk ratio where *P* < 0.05 is plotted so as not to obscure suggestive results but results with *P* > 0.05 are omitted for clarity. *Squares* of *dashed lines* indicate clusters of seronegative diseases (*upper left*) and seropositive (*lower right*). Diseases for which polygenic risk scores were calculated are listed horizontally while diseases for which an Icelandic cohort was available are listed vertically. *AS* Ankylosing spondylitis; *UC* ulcerative colitis; *CD* Crohn’s disease; *PSO* psoriasis; *MS* multiple sclerosis; *PBC*, primary biliary cirrhosis; *RA* rheumatoid arthritis; *SLE*, systemic lupus erythematosus; *T1D* type 1 diabetes mellitus; *Cel* celiac disease; *JIA* juvenile idiopathic arthritis

For eight ADs, the most predictive score was calibrated so that a unit increase in PRS dictated a doubling of risk of the corresponding disease (Online methods). Icelandic cohorts were not available for JIA and Cel so the scores were normalized to have a mean of zero and a standard deviation of one and a *P*-value threshold of 0.001 was arbitrarily selected. Next, we tested each PRS for association with nine other ADs and asthma, as an example of a non-autoimmune inflammatory disease. We found that ADs can be genetically divided into clusters of seronegative ADs (CD, UC, PSO and ankylosing spondylitis (AS)), in which autoantibodies are rarely seen, and seropositive ADs (JIA, RA, Cel, T1D, autoimmune thyroiditis (AITD) and systemic lupus erythematosus (SLE)), where autoantibodies are commonly found or represent characteristic features. Most diseases within each cluster show evidence of correlation but associations across the serological divide are rare (Fig. 3, Supplementary Table 8). Broadly speaking, this is the same trend as has been observed when comparing ADs on the basis of established risk loci.^13^

Interestingly, PBC and MS could not be placed in either cluster. The PBC-PRS associates with several diseases, both classified as seronegative and seropositive, while the MS-PRS only correlates strongly with PBC (Fig. 3). PBC-PRS corresponding to doubling in risk of PBC increased risk of MS by 29% (*P* = 4.3 × 10^−9^) and MS-PRS corresponding to doubling in risk of MS increased risk of PBC by 81% (*P* = 1.56 × 10^−7^). We also found that T1D-PRS more modestly associates with risk of MS but not vice versa. Several PRSs associated with PBC status, despite very modest size of this sample set, but no other PRSs than those for PBC and T1D associate with MS. We replicated these findings in the Swedish MS cohort (Table 2). We also explored the association of the PRSs with levels of disability, measured with the multiple sclerosis severity score (MSSS)^22^ in a subset of 5173 Swedish MS patients for which MSSS data were available (within the subset of samples that didn’t overlap with the IMSGC study, in the case of MS-PRS). Despite large sample size we observed no significant associations after correcting for multiple tests (Table 2), suggesting that MS severity is largely determined by factors other than those conferring genetic susceptibility to disease onset.

**Table 2:** Replication of primary biliary cirrhosis (PBC) and type 1 diabetes (T1D) polygenic risk score (PRS) prediction of multiple sclerosis (MS) in an independent Swedish cohort and results for the association of those PRSs and MS-PRS with the multiple sclerosis severity score (MSSS) after correcting for duration of disease

	MS-PRS	PBC-PRS	T1D-PRS
MS status	RR = 2.09 (1.98–2.20)	RR = 1.25 (1.21–1.29)	RR = 1.06 (1.02–1.10)
	*P* = 1.5 × 10^−165^	*P* = 3.1 × 10^−40^	*P* = 1.9 × 10^−3^
MSSS	*β* = 0.03(−0.01,0.07) MSSS units*	*β* = 0.03(−0.00,0.05) MSSS units	*β* = 0.03(0.00,0.06) MSSS units
	*P* = 0.11	*P* = 0.09	*P* = 0.04

Brackets enclose 95% confidence intervals for risk ratio estimates

*RR* risk ratio

*The MS PRS was tested for association with MS status and MSSS after excluding samples that overlapped with the IMSGC study (see text)

We note that the public data sets we used to derive the PRSs are based on the Immunochip, whose variants were selected to be more likely to associate with ADs than random variants, because these data sets represent the largest (or only) studies of these diseases for which summary statistics are available. Where possible, we repeated our analysis using studies covering the whole genome (excluding the MHC region) and obtained comparable results (Supplementary Tables 9–12).

### Proxy-phenotype analysis

Having established a strong genetic relationship between MS and PBC, we decided to use PBC as a proxy for MS. Out of 263 variants contributing to the PBC-PRS, 255 survived quality control in three or more MS data sets and 49 associate with MS after correction for multiple testing (*P* = 2 × 10^−4^). Out of the 49 significant variants 46 have concordant effects on MS and PBC (Supplementary Fig. 8 **)**. One of the discordant variants, rs10797431 at the *MMEL1* locus, has previously been reported to be genome-wide significant, both conferring risk of PBC^23^ and protection against MS.^2^ It has been shown that for ADs the most associated variant at a given locus frequently differs between the diseases and, even when shared, the same allele often has opposite effect.^13^ We excluded the variants with discordant effect from further analysis.

**Fig. 4:**
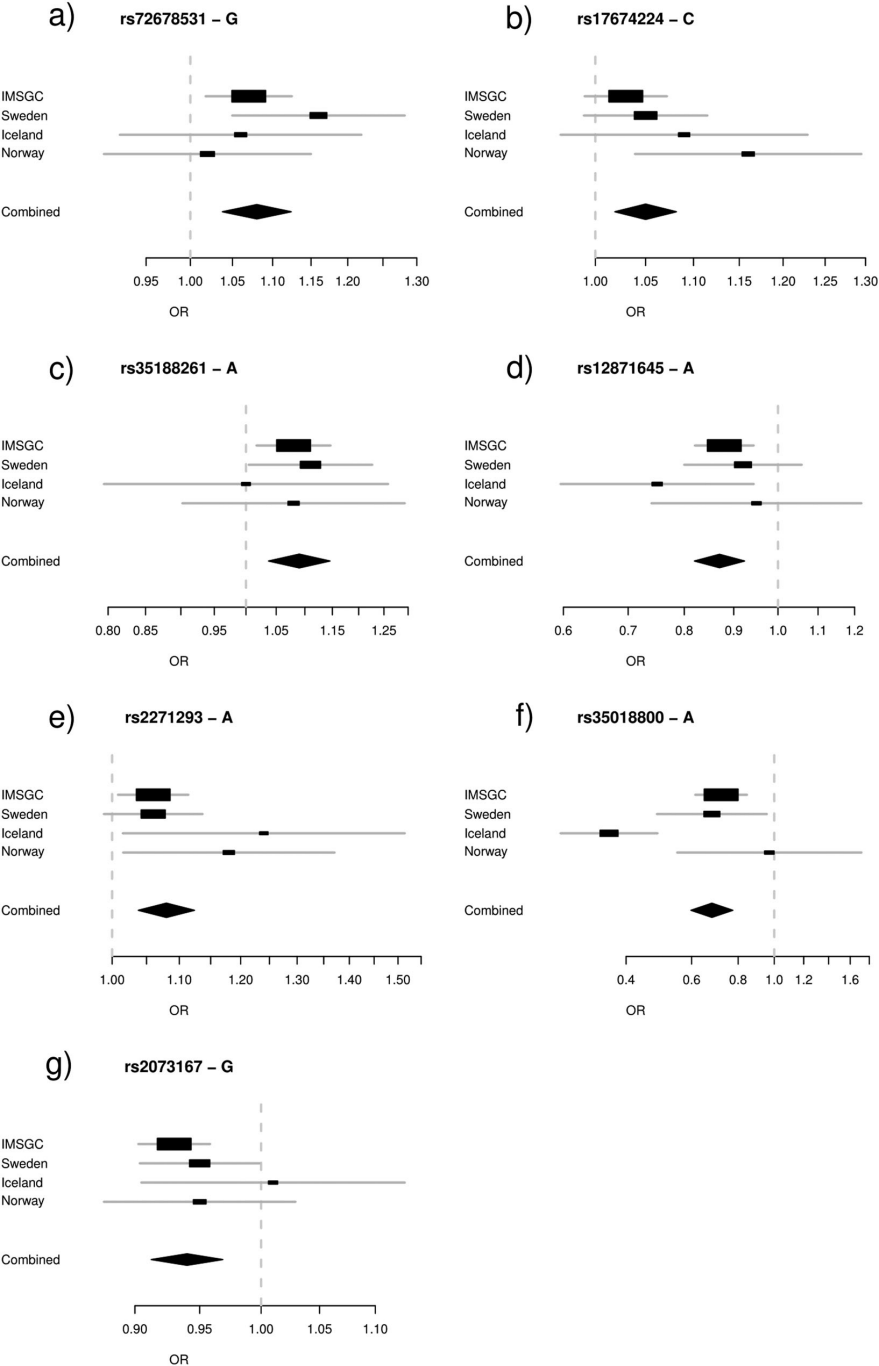
Forest plots for MS variants discovered through the proxy-phenotype method. **a** rs72678531; **b** rs17674224; **c** rs35188261; **d** rs12871645; **e** rs2271293; **f** rs35018800; **g** rs2073167

Out of the 46 remaining variants significantly associating with MS seven represent signals that are not explained by any of the 108 established MS variants (Table 3, Fig. 4, Supplementary Table 13 and Supplementary Figs. 9–15). rs35018800-A is a missense variant in tyrosine kinase 2 (*TYK2*), which causes a change of alanine to valine in position 928. This change, which has previously been reported to protect against RA and SLE,^24^ also protects against MS and is independent of the effect of another missense variant in TYK2, rs34536443-C, which has been shown to protect against MS.^25^

**Table 3:** Novel sequence variants that associate with MS identified by the proxy-phenotype method

						MS meta-analysis		PBC meta-analysis			
Chr	Position^a^	rsID	MA	OA	MAF [%]	*P*	OR (95% CI)	*P*	OR (95% CI)	Annotation	Closest Gene(s)
1	67332762	rs72678531	G	A	20.2	5.1 × 10^−6^	1.08 (1.04, 1.12)	2.5 × 10^−38^	1.61 (1.50, 1.74)	Intronic	*IL12RB2*
4	48075312	rs17674224	C	T	47.5	1.1 × 10^−4^	1.05 (1.02, 1.08)	4.8 × 10^−4^	1.11 (1.05, 1.18)	Intronic	*TXK, TEC*
7	129043485	rs35188261	A	G	13.9	4.2 × 10^−5^	1.09 (1.05, 1.14)	6.5 × 10^−22^	1.52 (1.39, 1.65)	Intronic	*TNPO3, IRF5*
13	50357429	rs12871645^b^	A	C	4.6	1.1 × 10^−4^	0.87 (0.81, 0.93)	3.3 × 10^−4^	0.77 (0.66, 0.89)	Intergenic	*DLEU1,DLEU1-AS1,ST13P4,DLEU2,MIR15A,MIR16-1*
16	67868167	rs2271293^c^	A	G	10.2	3.5 × 10^−5^	1.08 (1.04, 1.12)	5.1 × 10^−5^	1.20 (1.10, 1.31)	Upstream gene variant	*EDC4*
19	10354167	rs35018800^d^	A	G	1.4	9.2 × 10^−6^	0.68 (0.57, 0.81)	3.3 × 10^−4^	0.41 (0.25, 0.68)	Missense	*TYK2*
22	41395532	rs2073167	G	A	46.1	8.2 × 10^−6^	0.94 (0.91, 0.97)	4.0 × 10^−5^	0.88 (0.83, 0.94)	Intronic	*TEF*

The odds ratio (OR) reported is the OR of the minor allele

*MA* minor allele in Iceland, *OA* other allele, *MAF* minor allele frequency in Iceland, *MS* multiple sclerosis, *PBC* primary biliary cirrhosis, *CI* confidence interval

^a^ Build hg38

^b^ Conditioned on rs806321, found to associate with MS in the meta-analysis step

^c^ Conditioned on rs1886700, previously reported to associate with MS^2^

^d^ Conditioned on rs34536443, previously reported to associate with MS^25^

Many of the variants identified in the proxy-phenotype analysis may confer their risk through effects on interleukin signaling pathways. In addition to the missense variant in *TYK2*, rs17674224 is located in an intron of *TXK*, a gene that encodes another member of the tyrosine kinase family. TXK is a Th1-specific transcription factor and regulates the production of IFNg and other cytokines produced by these cells,^26^ important for specification and activation of various immune cells.

We also found that the G-allele of rs72678531, in an intron of *IL12RB2*, is associated with increased risk of MS. The encoded protein forms a subunit of IL-12 receptor, which upon binding of the cytokine IL-12 initiates an intracellular signaling cascade, involving several members of the tyrosine kinase family of proteins, ultimately leading to differentiation of naive T-cells into Th1 cells.^27^ The gene encoding the other subunit of the receptor, *IL12RB1*, has already been shown to associate with MS.^2^ We found that the G-allele rs72678531 is associated with increased expression of *IL12RB2* in whole blood (5.2% increase, *P* = 7.0 × 10^−15^) in the deCODE eQTL data set and in the GTEx data (*β* = 0.37 *SD*, *P* = 7.1 × 10^−13^). Finally rs35188261 is located in an intron of *TNPO3* close to *IRF5*, previously shown to associate with MS in a candidate gene study^28^ but has not reached significance in GWAS. The variant we report for this locus, rs35188261, shows low correlation (*r* ^2^ = 0.13) with the top variant from the candidate gene study of Kristjansdottir et al (rs4728142), and may represent a secondary signal in the locus if rs4728142 proves to be a true association. rs4728142 is not included in the PBC-PRS and came short of reaching genome-wide significance in the meta-analysis (*P* = 6.4 × 10^−7^). IRF5 regulates the transcription of type 1 interferon and other cytokines, including IL12.^29^ The beta interferons were the first disease-modifying therapies approved for MS, influencing the immune system at many levels, resulting in sustained anti-inflammatory state.^30^ We again refer to the supplementary note for discussion of all the newly identified variants.

## Discussion

We have discovered 14 variants not previously reported to associate with MS in GWAS. Seven of the variants were identified based on their association with PBC, the AD showing the strongest genetic relationship with MS (Table 3). Other than rs175126 in the *CD6, CD5* locus and rs35018800 in the *TYK2* locus, these variants are within loci not previously associated with MS. rs806321 and rs12871645 are two independent signals in the same novel locus. Most of the variants are within or close to immunologically relevant genes and many have previously been associated with other ADs (Supplementary note). Although only one variant could be directly associated with changes in transcription of close genes in whole blood, several of the variants are in regions that overlap with known binding sites for transcription factors and/or have histone modification profiles suggestive of possible enhancer function in GM cells from ENCODE (Supplementary note). We have postulated functional effects for these variants but further investigation is needed to establish their role in the pathogenesis of MS. The newly discovered variants explain 4.3% of the phenotypic variance in MS in Iceland compared to 22.9% explained by previously associated variants and 12.6% explained by HLA alleles showing additive association with disease in a recent publication.^31^ This estimate is conservative as it does not include dominant effects or gene–gene interactions identified in the region.

Two missense variants, both protecting against MS, were discovered in this study. The T-allele of rs1801133 (A222V missense) in *MTHFR* is known to increase the level of homocysteine in blood. The product of *MTHFR*, 5,10-methylenetetrahydrofolate reductase, catalyzes the conversion of 5,10-methylenetetrahydrofolate to 5-methyltetrahydrofolate, a co-substrate for vitamin B12-dependent homocysteine re-methylation to methionine. Vitamin B12 deficiency and MS share pathological changes and B12-dependent methylation and MTHFR have been suspected to play a role in MS for decades.^32^ Homocysteine levels in the blood and cerebrospinal fluid of MS patients have been found to be elevated in some studies,^33–35^ but not in others.^36, 37^ A recent study found different concentrations of methionine metabolites in brains of MS patients compared to brains of controls and suggested an effect on mitochondria and neuronal energetics.^38^

The variant rs35018800-A is a low-frequency (MAF = 1.4%, 0.77% and 0.97% in Iceland, Norway and Sweden, respectively) missense variant in *TYK2* and has the largest effect on MS risk of any variant outside the MHC region discovered to date. It is not clear what the effect of the amino acid substitution is on the function of the protein.

In the proxy-phenotype step, we observed strong enrichment and concordance of effect for PBC variants associating with MS (Supplementary Fig. 8). This is in agreement with 9.1% of the variants reported in the most recent IMSGC GWAS overlapping with PBC signals.^2^ However, the observed enrichment is likely to be also partially explained by the overlap in controls between the Immunochip studies of PBC and MS (Online methods). In the proxy-phenotype analysis, the best independent sequence variant for PBC in a region was selected and tested for association with MS. It is important to note that the best variant for PBC at a locus may not necessarily be the variant most strongly associated with MS (Supplementary Figs. 9 and 10). The same variant might be responsible for the signal observed in both studies but sampling noise determines precisely which variant in an linkage disequilibrium (LD) block tests most significant. As the IMSGC data include relatively few markers, it is also probable that neither of the top variants is the true causal variant responsible for the signal at the locus. We note that as the suggestive PBC variants associate significantly with MS, it seems probable that they truly associate with PBC as well. It is also important to note that although we applied genomic control (GC) to each of the Nordic cohorts, the IMSGC summary statistics have not been GC corrected, which may cause some inflation of test statistics. These variants survive correction for the number of tests performed. However, they do not reach the arbitrary but commonly used definition of genome-wide significance and some caution in their interpretation is warranted until they have been replicated by the scientific community.

We have used individual genotype data on a population level to comprehensively map genetic overlap between the most common ADs within a single population. We add to existing evidence of the polygenic architecture of these diseases and show that there is extensive genetic overlap between many pairs of ADs. In particular, we have established a strong genetic relationship between MS and PBC, and a less significant relationship between MS and T1D. A great number of the SNPs in and between genes so far associated with a variety of diseases are pleiotropic^39, 40^ and much work has been done to quantify the genetic overlap between traits.^41, 42^ This study establishes genetic relationships between many pairs of ADs and demonstrates how knowledge of genetic relationship can be used to establish priors and thereby to increase power of association studies, allowing for the identification of the specific pleiotropic loci responsible for the overlap. The sequence variants identified in this study explain a modest fraction of the phenotypic variance in MS but will hopefully help to identify biological pathways contributing to the disease.

Supplementary information is available at Genomic medicine’s website.

## Methods

All methods were performed in accordance with relevant guidelines and regulations.

### Subject recruitment

Icelandic samples used were obtained through ongoing deCODE studies of Icelanders. They were approved by the National Bioethics Committee (permit numbers: MS-VSN_15-212, RA-VSN_15-045, IBD-VSN_98-059, AS-VSN_98-020, PSO-VSN_14-118, T1D-VSN_12-156, AITD, SLE and PBC-VSN_08-059, Asthma-VSN-14-099) and the Icelandic Data Protection Authority. All patients and controls who donated DNA samples signed informed consent. Personal identifiers of the patient data and biological samples were encrypted by a third party system approved and monitored by the Data Protection Authority.

The Icelandic MS cohort consisted of patients diagnosed from 1950–2005 and followed-up at Landspitali, the National University Hospital of Iceland, or an outpatient department for MS patients in Reykjavik, Iceland.^43^ The RA patients were diagnosed in 1942–2010 at Landspitali, at the Centre for rheumatology research or at a private clinic in Reykjavik.^44^ RA was defined according to the 1987 revised criteria of the American college of rheumatology (ACR).^45^ All patients met four or more of the ACR criteria. Inflammatory bowel disease (IBD) patients consisted of all patients diagnosed with UC or CD by a gastroenterologist in Iceland 1950–2013. The diagnoses of all IBD patients were reviewed independently and fulfilled accepted diagnostic criteria, and all have had at least 1 year of follow-up evaluation and many patients have had decades of re-evaluation and confirmation of their final diagnosis.^46, 47^ The AS cohort consisted of all known AS patients in Iceland in 2010.^48^ All patients were interviewed and examined by a rheumatologist and found to fulfill the modified New York classification criteria for AS.^49^ PSO patients were diagnosed by dermatologists at Reykjavik dermatology clinic (1991–2014) or recruited through the Icelandic psoriasis association (SPOEX). A detailed clinical history was obtained by a structured questionnaire, and a careful physical examination was also carried out and the localization, distribution, and the size of the lesions were recorded.^50^ The T1D patients are all diagnosed with T1D and admitted to the national pediatric diabetes centre at Landspitali, for treatment and follow up until 2009.^51^ The AITD cohort consisted of all patients diagnosed with Grave’s disease or Hashimoto’s thyroiditis according to the ICD10 codes and ICD9 codes at Landspitali until 2010. Patients with systemic lupus erythomatosus (SLE) are all SLE patients diagnosed at the Landspitali, Centre for rheumatology research or at a private clinic of Reykjavik until 2011,^52^ who met at least four of the ACR criteria for the classification of SLE.^53^ Patients with PBC were identified at Landspitali by presence of anti-mitochondrial antibodies, ICD9 and ICD10 codes and pathological registries and the National death registry from 1991 to 2010.^54^

Asthma patients, 18–45 years of age, who visited an asthma clinic or emergency room at the National University Hospital of Iceland or the Icelandic Medical Centre (Laeknasetrid) during the years 1977–2014, received the ICD diagnosis or responded positively to the question: “Has a doctor confirmed your asthma diagnosis?” were included.^55^

The summary characteristics of the AD and asthma sample sets are available in Supplementary Table 5.

The Swedish MS cases and all but 2387 of the controls represent an extended collection of subjects from two population based case-control studies in Sweden, the Epidemiological investigation of multiple Sclerosis study and the Genes and environment in multiple Sclerosis study.^56^ All patients were diagnosed according to the McDonald criteria.^57^ Controls were randomly chosen from population registers and matched with cases by sex, age, and region of residence. The remaining 2387 controls are Swedish blood donors that were randomly ascertained from Skåne county in southern Sweden. Sample collection took place during summer and autumn of 2014. All samples were collected subject to ethical approval (Lund University ethical review board; dnr 2013/54).

The Norwegian MS cohort consists of 1013 MS patients recruited from the Oslo MS clinic and Norwegian MS biobank and registry. All patients were diagnosed according to the McDonald criteria.^57^ The MS biobank and registry also provided 30 controls. A further 23,333 controls come from three studies of Norwegians: 4856 samples constituted an extended set of Alzheimer’s and dementia subjects described previously,^58^ 6550 samples were recruited as a part of the Norwegian mother and child cohort study (MoBa),^59^ which includes more than 114,000 children, 95,000 mothers and 75,000 fathers. Pregnant women attending a routine ultrasound examination were initially invited. The first child was born in October 1999 and the last in July 2009. Further information can be found at [www.fhi.no/moba-en]. Finally 11,927 subjects come from the thematically organized psychosis research study. Subjects between ages 18–65 were recruited from psychiatric departments and outpatient clinics in Oslo. All studies were approved by the regional ethics committee and the Norwegian authorities for collection of medical data, and written informed consent was obtained from all participants.

### Genotyping, imputation and association analysis

Icelandic samples were genotyped on Illumina HumanHap300, HumanCNV30, HumanHap610, HumanHap1M, HumanHap660, Omni-1, Omni2.5 or Omni Express bead chips at deCODE Genetics. Prior to imputation, samples with <97% call rate were excluded as well as all SNPs with genotyping yield <95% or MAF < 1%. Some samples were genotyped on more than one chip and in those cases, all SNPs with substantial difference in call rate between chip types were excluded. Further, all SNPs showing *P* < 0.001 for deviation from Hardy–Weinberg equilibrium or a > 0.1% inheritance error rate were removed. Subjects were long range-phased and imputation into both chip-typed individuals and their close relatives was based on a panel of 8453 whole genome sequenced Icelanders.^60^ This process has been described in greater detail elsewhere.^60–62^ Briefly, regions of identity by descent are identified and used to phase haplotypes with great certainty. Making use of genealogy information, it is possible to deduce haplotypes for individuals that have not been genotyped, provided some of their relatives have been genotyped. Association testing was performed using logistic regression, adjusting for age and county of birth.

Genotyping of the Swedish cohort was carried out at deCODE using Illumina Omni chips. Phasing was performed using SHAPEIT2,^63, 64^ and imputation was carried out using IMPUTE2^65, 66^ based on the 1000 Genomes phase I integrated haplotypes generated using SHAPEIT2.^67^ Prior to imputation, SNPs having yield <95%, Hardy–Weinberg equilibrium *P*-values <1 × 10^−5^, or either A/T or G/C allele combinations were removed. Samples having <95% genotyping yield or evidence of non-European ancestry based on results from Structure using European (CEU), Chinese and Japanese (CHB + JPT) and Nigerian (YRI) individuals from the HapMap project as reference samples, as well as one of each pair of duplicate samples were also excluded. Association analysis was carried out using SNPTEST2^68^ with 20 principal components included as covariates. Principal components were calculated using EIGENSOFT.^69^

Genotyping of the Norwegian controls was carried out at deCODE using the Omni series of Illumina bead chips but Norwegian cases were genotyped on Human660-Quad at the Sanger institute in a collaboration with the International MS genetics consortium and the Wellcome Trust case control consortium.^70^ Samples were phased and imputed together based on the SNPs found on both chip types using the same methods and quality control as for the Swedish cohort. Association analysis was carried out using SNPTEST2^68^ with ten principal components included as covariates.

### Meta-analysis

We carried out an inverse-variance weighted meta-analysis under the assumption of fixed effect using the METAL software^71^ in two steps. First, we combined publicly available summary statistics from the largest study of MS to date, referred to as the IMSGC study, with summary statistics from our three Nordic cohorts. This resulted in combined statistics for 117,990 SNPs, which survived quality control in the IMSGC data and two or more of the Nordic cohorts. In the second step, we included in the Swedish cohort 1670 cases and 1534 controls that were excluded from the first analysis on the basis of overlap with the IMSGC study. Imputation and principal component calculations were repeated for the Swedish cohort after adding these samples. This resulted in combined summary statistics for 6,694,339 SNPs that survived quality control in all three Nordic cohorts.

Conditional analysis of the IMSGC data was performed using GCTA^72^ and the genotypes of 6500 randomly selected Icelanders as LD reference.

For some of the candidate markers, for example in the case of rs175126, the adjusted *P*-value was reported in Supplementary table 2 of the IMSGC paper.^2^ Where this information was available, we used the *P*-value provided by the IMSGC as that more accurately reflects the LD structure of all the study cohorts. Locus plots were generated using LocusZoom,^73^ displaying only variants surviving quality control in all cohorts (all Nordic cohorts only for rs1801133) and unconditioned *P*-values.

### Target and training sets for PRSs analyses

For PRS calculations, two types of data sets are required. The first, referred to as training set, comprises summary statistics from an external GWAS or a meta-analysis. The second, referred to as target set, consists of genotypes and phenotypes for a cohort that is independent of the training set cohort. For use as training sets, summary statistics from the discovery phase of many of the largest association studies of ADs to date were downloaded from http://www.immunobase.org/ (accessed 10.11.2015 by S.J.) and from https://www.ibdgenetics.org/downloads.html (accessed in January 2017 by S.O). Many of these studies have employed the Immunochip (an Illumina Infinum microarray) developed by the Immunochip consortium, to densely cover loci previously implicated in ADs.^4, 13^ For consistency, when only summary statistics from studies covering the whole genome were available, we extracted the SNPs found on the Immunochip and used only those SNPs for calculating the PRS. We later validated our findings using summary statistics from studies covering the whole genome.

From all training data sets, we drew the effect allele, the effect estimate and the *P*-value of the effect estimate and used those to calculate PRSs as described below. An overview of the respective studies is provided in Supplementary Table 3.

A sample of 150,656 genotyped Icelanders, representing more than half the Icelandic adult population, served as a target set. Subjects were drawn based on the following criteria:

1. Start with the genotypes of all 150,656 genotyped Icelanders.
2. Identify the age range of cases for each disease and include in the control group only people who fall within this age range.
3. Remove individuals who harbor long-range phased haplotypes found not to belong to the set of Icelandic haplotypes.
4. Remove individuals who have less than 98% probability of being of European ancestry based on results from Structure using genotypes for 2766 ethnicity-sensitive SNPs common to all Illumina SNP arrays and the HapMap CEU, CHB + JPT and YRI individuals as reference samples.^74^

For replication and for testing the association of PRSs with multiple sclerosis status scale (MSSS) we used the Swedish cohort previously described, excluding the blood bank donors from Skåne, which were added late to the study.

### Polygenic risk scores

PRSs were calculated based on the summary statistics of the training sets previously listed (Supplementary Table 4), excluding the extended MHC region (25–35 Mb of chromosome 6, build hg38) to ensure no variants in LD with the MHC region were included in the score.^16^ Markers found in the training data were matched with a set of in-house SNPs and only autosomal, biallelic SNPs with MAF > 1% and info > 0.9 in Iceland were included. We furthermore excluded AT/GC SNPs to avoid strand matching issues.

As variants within the MHC region show very strong association with all the diseases studied here, the exclusion of the MHC might be a source of controversy. However, its exclusion is critical to the study. PRSs can be used to establish biological pleiotropy by testing a score composed of a set of genetic variants contributing to the risk of a given trait for association with another. In this way, genetic overlap between traits can be detected, even in the absence of significantly associating signals.^42^ An underlying assumption is that the effect of a variant represents the effect of a single biological process common to both traits and variants are pruned so that only the variant showing the strongest evidence of association within a LD block is retained. However, due to extensive LD within the MHC region, the effect of a variant within that region is likely to be composed of the combined effect of several different genes on the disease. Some of these genes might contribute to both diseases while others will not. Excluding the MHC is therefore critical for avoiding the detection of spurious pleiotropy.

PLINK 1.9^75^ was used to prune SNPs in a sliding window of 500 kb, retaining the SNP which showed the strongest evidence of association with the phenotype in the training data and removed SNPs having *r* ^2^ > 0.1 with that SNP. A set of 960 whole genome sequenced Icelanders, unrelated at six meioses served as LD reference.^60^

We calculated a polygenic score for each individual, *j*, in the target data at ten different *P*-value inclusion thresholds using the formula

1 $$PR{S_j} = \mathop {\sum}\nolimits_{i \in S} {{\beta _i} \times {G_j}} ,$$

where *S* is the set of SNPs retained after pruning that have *P*-values below the inclusion threshold, *β* is the effect and *G*_*j*_ is the sum of the probability of the effect allele being found on either of individual’s *j* chromosomes. The pipeline used for calculating the PRS shares many features with the PRSice software.^76^

Each PRS, except those calculated for JIA and Cel, was tested for association with its corresponding disease in Iceland using generalized additive regression with smoothed age, sex and the first five principal components as covariates. The best *P*-value inclusion threshold was identified for each disease and the score at this threshold was calibrated so that a unit increase in the score represented a doubling in risk of its corresponding phenotype. This can be written as follows:

2 $${\rm{PRS}}\prime = \frac{{{\rm{PRS \times }}{\beta _{{\rm{PRS}}}}}}{{{\rm{log}}\left( 2 \right)}},$$

where PRS and PRS′ are the uncalibrated and calibrated polygenic risk scores, respectively, and *β*_PRS_ represents the log odds of the disease corresponding to the score in a logistic regression.

The calibrated score was then tested for association with disease status in each of the other target cohorts, using the same model as described above. Models were compared against null models that consisted of the covariates only and results were considered significant if *P* < 5.0 × 10^−4^.

For JIA and Cel, PRS were normalized to have a mean of 0 and a standard deviation of 1 in our sample of 150,656 Icelanders. As the most predictive threshold could not be determined, we arbitrarily selected the *P*-value inclusion threshold 0.001 and tested those scores for association with disease status in the same manner as described above. The un-calibrated PRSs were always normally distributed in the population and remained normally distributed after calibration and the use of these models is therefore justified. The ratio of the variance in PRS between cases and controls never exceeded 1.13 for case-lists with >500 cases and never exceeded 1.43 for case-lists with <500 cases.

Population stratification was estimated by randomly selecting 10,000 variants with minor allele frequency >5% from all over the genome and testing them for association with disease in each of the target cohorts. We calculated genomic inflation factor *λ* for the target phenotype in Iceland and adjusted the *P*-values of PRS-disease association accordingly. Nagelkerke’s pseudo-*R* ^2^ was used as a measure of the variance explained.

We tested the PRSs for MS, PBC and T1D for association with the MSSS using generalized additive regression with smoothed year of birth, gender and the first 20 principal components as covariates. The PRSs were standardized so that a unit increase corresponded to doubling in risk of the respective disease in Iceland. MSSS data were available for 5173 Swedish cases, 1466 of which overlapped with the IMSGC study. When testing the PBC-PRS and T1D-PRS for association with MSSS, all subjects were included but the overlapping samples were excluded before testing the association of the MS-PRS with MSSS.

### Proxy phenotype analysis

We extracted from the combined summary statistics from the meta-analysis 255 SNPs that contributed to the PRS for PBC and survived quality control in three or more MS data sets. Of those, all four study cohorts contributed to the statistics for 221 SNP. For these variants, a significance threshold of $2.0\, \times \,{10^{ - 4}} = \frac{{0.05}}{{255}}$ was applied. It is important to note that there was some sample overlap between the PBC study used to construct the PRS and the IMSGC discovery phase included in the meta-analysis. Out of 24,091 controls used in the IMSGC study, 4422 came from three sites within the United Kingdom, two of which also provided controls to the PBC study of Liu et al.^10^

Traditionally, only variants achieving genome-wide significance for phenotype A are tested in phenotype B in the proxy-phenotype method. Here we show that a substantial genetic signal is found among variants that have less significant *P*-values of association and take the novel approach to test all variants that make up the most predictive PRS for PBC for association with MS, including variants that do not reach genome-wide significance for PBC.

### Variance explained

The variance explained by previously identified and newly identified variants and HLA alleles was calculated using the formula:

3 $${R^2} = \mathop {\sum}\nolimits_{i \in A} {\beta _i^2 \times {f_i} \times \left( {1 - {f_i}} \right)} ,$$

where *β* is the log of the OR estimate for the variant and *f* is the frequency of the effect allele.

To estimate the variance explained by HLA alleles, we used the HLA alleles listed in Table 1 of a recent study of HLA alleles in MS.^31^ To facilitate comparison with the variants outside the MHC region, all of which were identified through an additive model of association, we used only those alleles reported to associate with MS in an additive model. In this way, our estimates are conservative.

### Annotation

We looked for overlap of our newly identified loci with other ADs by performing a literary search and by looking them up in Immunobase, a webpage containing most or all risk loci for ADs, available at http://www.immunobase.org/.

We performed an eQTL analysis in whole blood as previously described.^19^ Briefly, microarray hybridization assay was carried out on RNA isolated from blood of 1002 individuals and the expression intensities were adjusted for (age, neutrophil count, lymphocyte count, monocyte count)*sex. The *P*-values of association were generated by testing if the slope of the linear regression between expression and genotype was different from 0. These data are referred to as the deCODE in-house eQTL data. We also explored the effect of the variants on expression in data from the genotype expression trait (GTEx) project.^18^ As stated in the text, we only link the associated variant with changes in expression when it is either itself the variant most strongly associating with expression in the locus, or highly correlated (*r* ^2^ > 0.94) with the variant most strongly associating with expression in both data sets.

To determine if the variants overlapped TFBS we used the open regulatory annotation database (ORegAnno), which contains information on experimentally identified TFBS.^20^ To look for DNase I hypersensitivity clusters and regions with specific histone modifications, we used data from the ENCyclopedia of DNA elements (ENCODE) project.^21^ Although the precise mechanism by which histone modification regulates gene expression remains to be worked out, some patterns have emerged from recent research. These include that high levels of H3K4Me1 (single methylation on lysine residue number four of histone 3) but low levels of H3K4Me3 are indicative of an enhancer element while high H3K4Me3 characterizes promoter regions.^77^ Similarly, acetylation of lysine residue number 27 of histone 3 (H3K27ac) has been found to discriminate between active and inactive enhancer regions.^78^ We restricted our analysis to the seven cell lines included in the first phase of the ENCODE project but all results presented in this paper are from GM12878, which is a lymphoblastoid cell line and out of the seven, likely represents the most relevant cell type for the study of ADs. In our interpretation of these data, we are aware that although histone modification in promoters is relatively similar across cell types, the modification patterns in enhancers are often cell-type-specific.^79^ Therefore, even if our variants are found in regions that have histone modification pattern indicative of enhancer in GM12878, it is by no means certain that this is also the case in other cell types that might be more relevant in MS.

### Data availability

Summary statistics for ADs used to derive PRSs and, in the case of MS, included in a meta-analysis, are available at https://www.immunobase.org/ and https://www.ibdgenetics.org/. Other data are available from the corresponding authors upon reasonable request.

## Electronic supplementary material

Supplementary information

## References

[1] Olsson, T. & Piehl, F. in *Encyclopedia of Immunobiology* (ed. Ratcliffe, M. J. H.) 180–191 (Elsevier, 2016).

[2] International Multiple Sclerosis Genetics Consortium (IMSGC) (2013). Analysis of immune-related loci identifies 48 new susceptibility variants for multiple sclerosis. Nat. Genet..

[3] Cotsapas C (2011). Pervasive sharing of genetic effects in autoimmune disease. PLoS Genet..

[4] Cortes A (2010). Promise and pitfalls of the Immunochip. Arthritis Res. Ther..

[5] Eyre S (2012). High-density genetic mapping identifies new susceptibility loci for rheumatoid arthritis. Nat. Genet..

[6] Tsoi LC (2012). Identification of 15 new psoriasis susceptibility loci highlights the role of innate immunity. Nat. Genet..

[7] Trynka G (2011). Dense genotyping identifies and localizes multiple common and rare variant association signals in celiac disease. Nat. Genet..

[8] Onengut-Gumuscu S (2015). Fine mapping of type 1 diabetes susceptibility loci and evidence for colocalization of causal variants with lymphoid gene enhancers. Nat. Genet..

[9] Hinks A (2013). Dense genotyping of immune-related disease regions identifies 14 new susceptibility loci for juvenile idiopathic arthritis. Nat. Genet..

[10] Liu JZ (2012). Dense fine-mapping study identifies new susceptibility loci for primary biliary cirrhosis. Nat. Genet..

[11] Franke A (2010). Genome-wide meta-analysis increases to 71 the number of confirmed Crohn’s disease susceptibility loci. Nat. Genet..

[12] Anderson CA (2011). Meta-analysis identifies 29 additional ulcerative colitis risk loci, increasing the number of confirmed associations to 47. Nat. Genet..

[13] Parkes M, Cortes A, van Heel DA, Brown MA (2013). Genetic insights into common pathways and complex relationships among immune-mediated diseases. Nat. Rev. Genet..

[14] Rietveld CA (2014). Common genetic variants associated with cognitive performance identified using the proxy-phenotype method. Proc. Natl. Acad. Sci.

[15] The International Multiple Sclerosis Genetics Consortium (IMSGC). Evidence for polygenic susceptibility to multiple sclerosis—the shape of things to come. *Am. J. Hum. Genet*. **86**, 621–625 (2010). 10.1016/j.ajhg.2010.02.027 PMC2850422 20362272

[16] Stahl EA (2012). Bayesian inference analyses of the polygenic architecture of rheumatoid arthritis. Nat. Genet..

[17] Frosst P (1995). A candidate genetic risk factor for vascular disease: a common mutation in methylenetetrahydrofolate reductase. Nat. Genet..

[18] Lonsdale J (2013). The Genotype-Tissue Expression (GTEx) project. Nat. Genet..

[19] Emilsson V (2008). Genetics of gene expression and its effect on disease. Nature.

[20] Griffith OL (2007). ORegAnno: an open-access community-driven resource for regulatory annotation. Nucleic Acids Res..

[21] ENCODE Project Consortium (2004). T. E. P. The ENCODE (ENCyclopedia Of DNA Elements) Project. Science.

[22] Roxburgh RHSR (2005). Multiple Sclerosis Severity Score: using disability and disease duration to rate disease severity. Neurology.

[23] Hirschfield GM (2010). Variants at IRF5-TNPO3, 17q12-21 and MMEL1 are associated with primary biliary cirrhosis. Nat. Genet..

[24] Diogo D (2015). TYK2 protein-coding variants protect against rheumatoid arthritis and autoimmunity, with no evidence of major pleiotropic effects on non-autoimmune complex traits. PLoS One.

[25] Mero I-L (2010). A rare variant of the TYK2 gene is confirmed to be associated with multiple sclerosis. Eur. J. Hum. Genet..

[26] Takeba Y, Nagafuchi H, Takeno M, Kashiwakura J, Suzuki N (2002). Txk, a member of nonreceptor tyrosine kinase of Tec family, acts as a Th1 cell-specific transcription factor and regulates IFN-gamma gene transcription. J. Immunol..

[27] Hsieh CS (1993). Development of TH1 CD4+T cells through IL-12 produced by Listeria-induced macrophages. Science.

[28] Kristjansdottir G (2008). Interferon regulatory factor 5 (IRF5) gene variants are associated with multiple sclerosis in three distinct populations. J. Med. Genet..

[29] Takaoka A (2005). Integral role of IRF-5 in the gene induction programme activated by Toll-like receptors. Nature.

[30] Severa M, Rizzo F, Giacomini E, Salvetti M, Coccia EM (2015). IFN-β and multiple sclerosis: cross-talking of immune cells and integration of immunoregulatory networks. Cytokine Growth Factor Rev..

[31] Moutsianas L (2015). Class II HLA interactions modulate genetic risk for multiple sclerosis. Nat. Genet..

[32] Huang WX, He B, Hillert J (1997). A methylenetetrahydrofolate reductase gene polymorphism in multiple sclerosis. Eur. J. Neurol.

[33] Vrethem M (2003). Increased plasma homocysteine levels without signs of vitamin B12 deficiency in patients with multiple sclerosis assessed by blood and cerebrospinal fluid homocysteine and methylmalonic acid. Mult. Scler..

[34] Ramsaransing GSM (2006). Plasma homocysteine levels in multiple sclerosis. J. Neurol. Neurosurg. Psychiatry.

[35] Kocer B, Engur S, Ak F, Yılmaz M (2009). Serum vitamin B12, folate, and homocysteine levels and their association with clinical and electrophysiological parameters in multiple sclerosis. J. Clin. Neurosci..

[36] Kararizou E (2013). Plasma homocysteine levels in patients with multiple sclerosis in the Greek population. J. Chinese Med. Assoc..

[37] Rio J (1994). Serum homocysteine levels in multiple sclerosis. Arch. Neurol..

[38] Singhal NK (2015). Changes in methionine metabolism and histone H3 trimethylation are linked to mitochondrial defects in multiple sclerosis. J. Neurosci..

[39] Solovieff N, Cotsapas C, Lee PH, Purcell SM, Smoller JW (2013). Pleiotropy in complex traits: challenges and strategies. Nat. Rev. Genet..

[40] Sivakumaran S (2011). Abundant pleiotropy in human complex diseases and traits. Am. J. Hum. Genet..

[41] Bulik-Sullivan B (2015). An atlas of genetic correlations across human diseases and traits. Nat. Genet..

[42] International Schizophrenia Consortium (2009). Common polygenic variation contributes to risk of schizophrenia and bipolar disorder. Nature.

[43] Benedikz J (2002). The natural history of untreated multiple sclerosis in Iceland. A total population-based 50 year prospective study. Clin. Neurol. Neurosurg..

[44] Grant SF (2001). The inheritance of rheumatoid arthritis in Iceland. Arthritis Rheum..

[45] Arnett FC (1988). The American Rheumatism Association 1987 revised criteria for the classification of rheumatoid arthritis. Arthritis Rheum..

[46] Reynisdottir I (2004). A genetic contribution to inflammatory bowel disease in Iceland: A genealogic approach. Clin. Gastroenterol. Hepatol..

[47] Björnsson S, Jóhannsson JH (2000). Inflammatory bowel disease in Iceland, 1990-1994: a prospective, nationwide, epidemiological study. Eur. J. Gastroenterol. Hepatol..

[48] Geirsson AJ, Eyjolfsdottir H, Bjornsdottir G, Kristjansson K, Gudbjornsson B (2010). Prevalence and clinical characteristics of ankylosing spondylitis in Iceland—a nationwide study. Clin. Exp. Rheumatol..

[49] van der Linden S, Valkenburg HA, Cats A (1984). Evaluation of diagnostic criteria for ankylosing spondylitis. A proposal for modification of the New York criteria. Arthritis Rheum..

[50] Karason A (2005). Genetics of psoriasis in Iceland: evidence for linkage of subphenotypes to distinct loci. J. Invest. Dermatol..

[51] Hanberger L (2014). Childhood diabetes in the Nordic countries: a comparison of quality registries. J. Diabetes Sci. Technol.

[52] Kristjansdottir H (2008). Association of three systemic lupus erythematosus susceptibility factors, PD-1.3A, C4AQ0, and low levels of mannan-binding lectin, with autoimmune manifestations in Icelandic multicase systemic lupus erythematosus families. Arthritis Rheum..

[53] Tan EM (1982). The 1982 revised criteria for the classification of systemic lupus erythematosus. Arthritis Rheum..

[54] Baldursdottir TR (2012). The epidemiology and natural history of primary biliary cirrhosis: a nationwide population-based study. Eur. J. Gastroenterol. Hepatol..

[55] Gudbjartsson DF (2009). Sequence variants affecting eosinophil numbers associate with asthma and myocardial infarction. Nat. Genet..

[56] Hedström AK, Hillert J, Olsson T, Alfredsson L (2014). Alcohol as a modifiable lifestyle factor affecting multiple sclerosis risk. JAMA Neurol..

[57] Polman CH (2011). Diagnostic criteria for multiple sclerosis: 2010 Revisions to the McDonald criteria. Ann. Neurol..

[58] Steinberg S (2015). Loss-of-function variants in ABCA7 confer risk of Alzheimer’s disease. Nat. Genet..

[59] Magnus P (2006). Cohort profile: the Norwegian Mother and Child Cohort Study (MoBa). Int. J. Epidemiol..

[60] Gudbjartsson DF (2015). Large-scale whole-genome sequencing of the Icelandic population. Nat. Genet..

[61] Styrkarsdottir U (2013). Nonsense mutation in the LGR4 gene is associated with several human diseases and other traits. Nature.

[62] Kong A (2008). Detection of sharing by descent, long-range phasing and haplotype imputation. Nat. Genet..

[63] Delaneau O, Marchini J, Zagury J-F (2011). A linear complexity phasing method for thousands of genomes. Nat. Methods.

[64] Delaneau O, Zagury J-F, Marchini J (2012). Improved whole-chromosome phasing for disease and population genetic studies. Nat. Methods.

[65] Howie BN, Donnelly P, Marchini J (2009). A Flexible and Accurate Genotype Imputation Method for the Next Generation of Genome-Wide Association Studies. PLoS Genet..

[66] Howie B, Marchini J, Stephens M (2011). Genotype imputation with thousands of genomes. G3 (Bethesda).

[67] Auton A (2015). A global reference for human genetic variation. Nature.

[68] Marchini J, Howie B, Myers S, McVean G, Donnelly P (2007). A new multipoint method for genome-wide association studies by imputation of genotypes. Nat. Genet..

[69] Price AL (2006). Principal components analysis corrects for stratification in genome-wide association studies. Nat. Genet..

[70] International Multiple Sclerosis Genetics Consortium (2011). Genetic risk and a primary role for cell-mediated immune mechanisms in multiple sclerosis. Nature.

[71] Willer CJ, Li Y, Abecasis GR (2010). METAL: fast and efficient meta-analysis of genomewide association scans. Bioinformatics.

[72] Yang, J., Lee, S. H., Goddard, M. E. & Visscher, P. M. GCTA: a tool for genome-wide complex trait analysis. *Am. J. Hum. Genet*. **88**, 76–82 (2011). 10.1016/j.ajhg.2010.11.011 PMC3014363 21167468

[73] Pruim RJ (2010). LocusZoom: regional visualization of genome-wide association scan results. Bioinformatics.

[74] International T (2003). The International HapMap Project. Nature.

[75] Purcell, S. *et al*. PLINK: a tool set for whole-genome association and population-based linkage analyses. *Am. J. Hum. Genet*. **81**, 559–575 (2007). 10.1086/519795 PMC1950838 17701901

[76] Euesden J, Lewis CM, O’Reilly PF (2015). PRSice: Polygenic Risk Score software. Bioinformatics.

[77] Heintzman ND (2007). Distinct and predictive chromatin signatures of transcriptional promoters and enhancers in the human genome. Nat. Genet..

[78] Creyghton MP (2010). Histone H3K27ac separates active from poised enhancers and predicts developmental state. Proc. Natl. Acad. Sci. USA.

[79] Heintzman ND (2009). Histone modifications at human enhancers reflect global cell-type-specific gene expression. Nature.

