# Supplementary material for: Fourteen sequence variants that associate with multiple sclerosis discovered by meta-analysis informed by genetic correlations
Source: NPJ Genom Med. 2017 Aug 8;2:24. doi: 10.1038/s41525-017-0027-2 (PMC5677966; doi:10.1038/s41525-017-0027-2)
Supplement: Supplementary file 1 — Supplementary information [file 41525_2017_27_MOESM1_ESM.pdf]

Supplementary information

The supplementary information includes:

Supplementary Note on variant annotation and the function of the gene closest to each of the variants found to associate with MS in this study

Thirteen supplementary tables containing cohort information, association results and the number of variants making up each polygenic risk score.

Fourteen locus plots of newly discovered variants and one q-q plot.

References cited are at the end of the document.

Supplementary Note. Variant annotation and the function of the gene closest to each of the variants identified to associate with MS in this study

1. **Variants identified in the meta-analysis stage:**

rs1801133 is a missense variant in the 5,10-methylenetetrahydrofolate reductase gene (*MTHFR),* a key enzyme in the intracellular folate (vitamin B9) metabolism and homeostasis. It is known to increase homocysteine levels (see text) and the T-allele has been established as a risk allele for coronary heart disease[1] but has been found to be protective for Graves' disease[2]. Vitamin B12-dependent methionine metabolism is dysregulated in the MS brain (see text).

rs9427431 is an intergenic variant close to protein tyrosine phosphatase, receptor type, c (*PTPRC*), also known as antigen CD45. This protein is heavily expressed on the surface of T and B lymphocytes and is essential for T-cell receptor signaling and T-cell activation[3]. A point mutation in exon 4 of this gene has previously been associated with MS[4] but this has failed to replicate in other studies[5,6].

rs11707807 is located in an intron of the lipoma preferred partner (*LPP*) gene at a locus which has been associated with Cel[7], AITD[8] and Vitiligo[9]. The specific variant is found within the binding site of the insulator binding protein CCCTC-binding factor (CTCF), but no effect on expression of close genes was detected.

rs13260060 is located in the intron of the nuclear receptor coactivator 2 gene (*NCOA2*). It is not in a DNase I hypersensitivity cluster and no histone modification profile indicative of a particular regulatory element was found in the ENCODE data. The encoded protein of *NCOA2* functions as a transcriptional coactivator for all steroid receptors and with many class II nuclear receptors, such as thyroid, retinoic acid, retinoid X and vitamin D receptors[10–12].

rs175126 is as secondary signal in the *CD5,CD6* locus. It is intergenic and is located in a region with high H3K4Me1 but little H3K27Ac in GM12878 cells, the profile indicative of inactive enhancer regions[13]. It also overlaps a binding site for the transcription factor early growth response-1 (*EGR1*). No significant association with the expression of any gene was found.

rs4245080 is located in the intron of ETS proto-oncogene 1 (*ETS1*). The encoded protein is a transcription factor known to play a central role in T-cell maturation and development in the thymus. Specifically, it promotes the expression of runt related transcription factor 3 (*RUNX3*) and is required for the development of CD8+ T-cells[14]. ETS1 is also a negative regulator of Th17 cells and knock-out mice have increased levels of Th17 cells and high levels of IL-17 as a result[15]. Variants in *ETS1* have been associated with RA, PSO, SLE and Cel[7,16–18]. rs4245080 is found within a DNase I hypersensitivity cluster in a region with high levels of H3K4Me1 and H3K27Ac in GM12878 cells in the ENCODE data, but this histone modification profile has been found to be indicative for active enhancer regions[19].

rs806321 is located within a DNase I hypersensitivity cluster in an intron of the deleted in lymphocytic leukemia 1 (*DLEU1*) gene. This gene doesn't encode a protein but encodes a long non-coding RNA (lncRNA) molecule.

2. Variants identified by the proxy-phenotype method:

rs72678531 is found within an intron of *IL12RB2*. The encoded protein forms a subunit of a cytokine IL-12 receptor that is important for the differentiation of naive T-cells into Th1 cells[20]. Variants in the gene have been reported to associate with PBC[21], as well as Bechet's disease[22], systemic sclerosis[23], UC[24] and CD[24,25]. Furthermore, *IL12RB2*(-/-) mice have been shown to be at increased risk of developing experimental autoimmune encephalomyelitis, a murine model for MS[26]. rs72678531 is in a region with high H3K4Me1 and high H3K27Ac in GM12878 cells. The variant associates with changes in transcription of *IL12RB2* in whole blood (see text).

rs17674224 is within an intron of (*TXK)*, which encodes TXK tyrosine kinase. Tyrosine kinases strongly influence gene expression and differentiation of immune cells and participate in regulation of immune response. However, variants in *TXK* have so for only been associated with Crohn's disease[24] in GWAS, although excessive expression of TXK has also been found in patients with Bechet's disease[27].

rs35188261 is located in an intron of transportin 3 (*TNPO3*) 3' downstream of *IRF5*. In addition to PBC, variants in the locus have been reported to associate with SLE[28], RA[29],Sjörgen syndrome[30], systemic sclerosis[31], CD[24] and UC[24]. The potential role of TNPO3 in autoimmunity is not clear but IRF5 is a transcription factor that regulates the production of multiple cytokines. Another member of this family of transcription factors, IRF8, has previously been associated with the risk of MS[32]. rs35188261 is in a region with high H3K4Me1 but little H3K27Ac.

rs12871645 is intergenic found in the same region as rs806321 but remains significant after conditioning on that variant. High levels of H3K4Me1 but no H3K27Ac are found in the region in GM12878 cells in ENCODE, which indicates that there may be an inactive enhancer in the region[19]. No evidence of association with expression was found.

rs2271293 is found upstream of the enhancer of mRNA decapping 4 (*EDC4*) gene and represents a secondary signal in the locus. The encoded protein is a subunit of a complex involved in the decapping of mRNA after its transfer from the nucleus. This decapping complex plays a role in immune response through post-transcriptional regulation of various cytokines and chemokines[33]. The risk allele (A) for MS and PBC has also been associated with decreased high-density lipoprotein (HDL) concentration in blood[34]. rs2271293 is in complete LD with the missense variant rs73594554 in neuritin 1 like (*NRN1L*).

rs35018800 is a missense variant in tyrosine kinase 2 (*TYK2*) and causes alanine in positon 928 to be changed into valine. Tyrosine kinases play central roles in intracellular signaling following cytokine binding to extracellular domains of membrane bound receptors but the effect of this substitution on the function of the enzyme (if any) is unclear. Variants in *TYK2* have been associated with MS as well as almost all other common ADs to date[17,21,24,29,32,35–37].

rs2073167, lies within an intron of the thyrotroph embryonic factor (*TEF*) gene that encodes a member of the proline and acidic amino acid-rich (PAR) superfamily of leucine zipper transcription factors[38]. The locus has previously been linked with Vitiligo[9].

Supplementary Table 1: Multiple sclerosis case-control cohorts used in the meta-analysis.

	**Nr of cases**	**Nr of controls**	**λ**
IMSGC study	14,498	24,091	-
Swedish cohort	4,505	6,105	1.07
Swedish cohort including IMSGC overlapping samples	6,175	7,639	1.10
Icelandic cohort	1,063	317,639	1.10
Icelandic cohort - genotyped	774	149,882	-
Norwegian cohort	1,013	23,363	1.04
**Total excluding IMSGC overlap**	**21,079**	**371,198**	

Supplementary Table 2: Association statistics for SNPs reaching genome-wide significance in a meta-analysis in each cohort individually. Effect allele (and minor allele) is enclosed in brackets. *Neither the variant nor a good proxy is included on the immunochip. Swedish association statistics are from an analysis of 6,175 cases and 7,639 controls.

	IMSGC meta-analysis		Swedish cohort		Icelandic cohort		Norwegian cohort	
**rsID**	**P**	**OR**	**P**	**OR**	**P**	**OR**	**P**	**OR**
rs1801133* (T)	NA	NA	8.9×10^{-7}	0.87 (0.82, 0.92)	0.063	0.91 (0.82, 1.01)	0.012	0.88 (0.80, 0.97)
rs9427431 (C)	1.1×10^{-4}	0.94 (0.91, 0.97)	0.0013	0.91 (0.86, 0.96)	0.099	0.92 (0.83, 1.02)	0.046	0.91 (0.83, 1.00)
rs11707807 (G)	2.2×10^{-5}	1.07 (1.04, 1.11)	0.0019	1.10 (1.03, 1.16)	0.14	1.08 (0.98, 1.19)	0.072	1.08 (0.99, 1.18)
rs13260060 (A)	5.9×10^{-6}	1.13 (1.07, 1.19)	0.011	1.13 (1.03, 1.23)	0.24	1.11 (0.93, 1.31)	0.049	1.13 (1.00, 1.27)
rs175126 (G)	3.0×10^{-5}	1.07 (1.04, 1.11)	2.0×10^{-7}	1.19 (1.12, 1.26)	0.0074	1.15 (1.04, 1.26)	0.018	1.11 (1.02, 1.20)
rs4245080 (A)	0.00095	1.06 (1.03, 1.11)	0.00060	1.11 (1.04, 1.17)	0.0046	1.15 (1.04, 1.27)	0.048	1.10 (1.00, 1.20)
rs806321 (C)	8.0×10^{-6}	0.93 (0.90, 0.96)	0.00069	0.90 (0.85, 0.96)	0.05	0.93 (0.86, 1.00)	0.019	0.89 (0.81, 0.98)

Supplementary Table 3: Number of sequence variants closer than 500 kb to our reported variant.

rsID	$r^2 > 0.9$	$r^2 > 0.5$	Coding or splice site variants
rs1801133	0	3	0
rs11707807	36	87	0
rs13260060	115	197	0
rs175126	3	4	0
rs4245080	6	29	0
rs9562970	72	107	0
rs806321	9	56	0
rs72678531	22	54	0
rs17674224	23	70	0
rs35188261	45	103	0
rs12871645	44	57	0
rs2271293	33	414	rs73594554 (r^2=1)
rs35018800	0	14	0
rs2073167	1	46	0

Supplementary Table 4: Overview of studies from which summary statistics were used to calculate polygenic risk scores (training sets).

Phenotype	# cases	# controls	Reference
Juvenile idiopathic arthritis	772	8,530	[35], UK cohort only
Celiac disease	12,041	12,228	[7]
Primary Biliary cirrhosis	2,861	8,514	[21]
Type 1 diabetes	10,796	12,173	[37]
Psoriasis	2,997	9,183	[17], GAPC cohort only
Multiple Sclerosis	14,498	24,091	[32]
Rheumatoid arthritis	11,475	15,870	[29]
Systemic Lupus Erythematosus	4,036	6,959	[18]
Crohn's disease	14,594	26,715	[39]
Ulcerative colitis	10,679	26,715	[39]

For Systemic lupus erythematosus (SLE) only a study covering the whole genome was available. PRS was only calculated for SNPs found on the immunochip.

Supplementary Table 5: Summary characteristics of the Icelandic and Swedish target cohorts. RA: Rheumatoid arthritis; MS: Multiple sclerosis; SD: Standard deviation.

Disease	# cases (Female)	# controls (Female)	Mean age [years] (case/control)	SD age [years] (case/control)
Ankylosing spondylitis	298 (115)	133,101 (71,113)	54.43/55.25	12.52/18.13
Autoimmune thyroiditis	916 (753)	139,574 (74,434)	55.61/55.66	16.12/19.49
Crohn‘s disease	262(142)	137,444 (73,732)	55.12/56.40	17.49/18.95
Multiple Sclerosis	358 (255)	130,086 (70,029)	59.16/58.69	13.46/17.75
Psoriasis	4,343 (2,405)	137,376 (73,535)	58.02/55.76	16.63/19.85
Primary Biliary cirrhosis	110 (95)	117,522 (63,069)	70.8/61.19	11.67/15.63
Rheumatoid arthritis	1,069 (823)	127,776 (68,551)	67.88/58.92	14.56/17.45
Seropositive RA	599 (470)	128,247 (68,904)	68.49/58.95	14.34/17.45
Seronegative RA	333 (251)	124,402 (66,992)	67.14/60.00	14.77/16.78
Systemic Lupus	225 (206)	132,623 (71,018)	61.93/56.80	15.83/18.04
Type 1 diabetes	462 (225)	140,190 (74,909)	43.95/55.41	18.75/19.52
Ulcerative colitis	1,120 (522)	134,362 (72,460)	59.98/57.54	16.40/18.66
Asthma	1,843 (1,195)	47,298 (25,343)	34.98/33.76	7.92/8.63
Swedish MS cohort	6,281 (4,542)	5,330 (4,003)	55.30/55.53	13.44/13.24

Supplementary Table 6: Summary of most predictive thresholds when PRS were tested against their corresponding phenotype in an independent target set. For JIA and Cel, no target data was available and the P-value inclusion threshold was arbitrarily selected as 0.001. *The SLE study used here did not use the immunochip. For this study, variants of the immunochip were extracted and used for calculating the scores. We report Nagelkerke's R^2 as a measure of the variance explained.

Training phenotype	Pt	R^2 (%)	P
Juvenile idiopathic arthritis	1×10^{-3}	NA	NA
Celiac	1×10^{-3}	NA	NA
Primary Biliary cirrhosis	1×10^{-3}	4.0	9.5×10^{-16}
Type 1 diabetes	0.1	4.3	6.1×10^{-51}
Psoriasis	0.5	0.25	1.5×10^{-15}
Multiple Sclerosis	0.01	2.8	4.1×10^{-33}
Rheumatoid arthritis	0.01	0.59	2.5×10^{-15}
Systemic lupus erythematosus*	0.4	3.6	9.4×10^{-24}
Crohn's disease	1×10^{-3}	3.6	2.3×10^{-30}
Ulcerative colitis	1×10^{-3}	3.9	1.2×10^{-93}

Supplementary Table 7: Number of variants making up each polygenic risk score based on variants found on the immunochip and passing selection criteria. The most predictive threshold for each disease is shown in bold. MS: Multiple sclerosis; Cel: Celiac disease; Jia: Juvenile idiopathic arthritis; PBC: Primary biliary cirrhosis; PSO: Psoriasis; T1D: Type 1 diabetes; RA: Rheumatoid arthritis; SLE: Systemic lupus erythematosus; CD: Crohn's disease; UC: Ulcerative colitis.

Phenotype	1×10^{-6}	1×10^{-5}	1×10^{-4}	1×10^{-3}	0.01	0.1	0.2	0.3	0.4	0.5
MS	75	117	183	328	**879**	3,947	6,559	8,898	11,146	13,257
Cel	73	98	163	**342**	952	4,093	6,582	8,686	10,643	12,370
Jia	2	5	24	**89**	495	3,278	5,870	8,159	10,305	12,211
PBC	42	59	112	**263**	898	4,168	6,989	9,310	11,443	13,386
PSO	91	129	203	383	1,045	4,462	7,125	9,437	11,524	**13,410**
T1D	81	114	199	380	1,063	**4,322**	6,976	9,251	11,310	13,166
RA	28	42	81	176	**713**	3,957	6,727	9,023	11,101	13,043
SLE	49	68	112	262	893	4,082	6,745	9,015	**11,085**	12,884
CD	315	420	623	**1,020**	2,007	5,627	8,181	10,248	12,053	13,738
UC	203	284	444	**769**	1,718	5,373	7,978	10,134	12,022	13,763

Supplementary Table 8: Results from polygenic risk score analysis. Risk ratios and 95% confidence intervals for each disease pair with P-values shown in italic below. Diseases for which we calculated polygenic risk score are listed horizontally while diseases for which an Icelandic cohort was available are listed vertically.

Disease	UC	CD	PSO	MS	PBC	Cel	T1D	JIA	RA	SLE
**AS**	1.31 (1.14, 1.50)<br>9.1×10^{-5}	1.25 (1.10, 1.42)<br>4.7×10^{-4}	0.87 (0.48, 1.59)<br>*0.65*	0.97 (0.85, 1.11)<br>*0.66*	1.02 (0.90, 1.15)<br>*0.75*	1.14 (1.01, 1.29)<br>*0.039*	1.01 (0.90, 1.13)<br>*0.86*	1.07 (0.94, 1.21)<br>*0.29*	1.26 (0.90, 1.77)<br>*0.18*	1.00 (0.90, 1.11)<br>*0.96*
**AITD**	1.02 (0.96, 1.09)<br>*0.54*	0.97 (0.88, 1.07)<br>*0.54*	1.28 (0.93, 1.76)<br>*0.13*	1.04 (0.97, 1.11)<br>*0.25*	1.12 (1.06, 1.19)<br>1.4×10^{-4}	1.14 (1.06, 1.22)<br>1.7×10^{-4}	1.25 (1.18, 1.33)<br>4.7×10^{-13}	1.16 (1.09, 1.24)<br>1.3×10^{-5}	1.62 (1.36, 1.93)<br>6.2×10^{-8}	1.14 (1.07, 1.22)<br>7.7×10^{-5}
**CD**	1.75 (1.54, 1.99)<br>3.0×10^{-17}	2.00 (1.78, 2.25)<br>2.8×10^{-30}	1.40 (0.77, 2.55)<br>*0.27*	1.10 (0.95, 1.27)<br>*0.20*	1.07 (0.97, 1.18)<br>*0.19*	1.00 (1.00, 1.00)<br>*0.99*	1.06 (0.94, 1.19)<br>*0.33*	1.12 (0.99, 1.27)<br>*0.079*	1.18 (0.86, 1.62)<br>*0.31*	0.94 (0.83, 1.06)<br>*0.28*
**MS**	1.12 (1.01, 1.24)<br>*0.03*	1.04 (0.95, 1.14)<br>*0.39*	1.85 (1.14, 2.99)<br>*0.012*	2.00 (1.79, 2.24)<br>4.1×10^{-33}	1.29 (1.18, 1.40)<br>4.3×10^{-9}	0.98 (0.88, 1.09)<br>*0.72*	1.18 (1.08, 1.30)<br>4.9×10^{-4}	1.01 (0.91, 1.12)<br>*0.85*	1.16 (0.89, 1.51)<br>*0.27*	1.09 (0.98, 1.20)<br>*0.10*
**PBC**	1.17 (0.95, 1.43)<br>*0.13*	1.12 (0.93, 1.35)<br>*0.24*	1.53 (0.60, 3.88)<br>*0.37*	1.81 (1.45, 2.26)<br>1.6×10^{-7}	2.00 (1.69, 2.37)<br>9.5×10^{-16}	1.29 (1.06, 1.57)<br>9.9×10^{-3}	1.05 (0.89, 1.24)<br>*0.56*	1.17 (0.97, 1.42)<br>*0.11*	1.90 (1.13, 3.19)<br>*0.015*	1.54 (1.27, 1.86)<br>1.0×10^{-5}
**PSO**	1.11 (1.07, 1.15)<br>3.0×10^{-8}	1.13 (1.09, 1.17))<br>6.9×10^{-13}	2.00 (1.69, 2.37)<br>1.5×10^{-15}	1.05 (1.01, 1.09)<br>*0.011*	1.08 (1.05, 1.11)<br>1.9×10^{-7}	1.10 (1.06, 1.14)<br>1.1×10^{-7}	1.04 (1.01, 1.07)<br>*0.020*	1.06 (1.02, 1.10)<br>2.3×10^{-3}	1.06 (0.97, 1.16)<br>*0.19*	0.97 (0.94, 1.00)<br>*0.087*
**RA**	1.02 (0.97, 1.08)<br>*0.47*	1.02 (0.95, 1.09)<br>*0.56*	0.99 (0.79, 1.24)<br>*0.93*	1.04 (0.97, 1.12)<br>*0.28*	1.10 (1.04, 1.17)<br>1.3×10^{-3}	1.13 (1.06, 1.20)<br>1.9×10^{-4}	1.13 (1.07, 1.20)<br>4.6×10^{-5}	1.13 (1.06, 1.21)<br>2.3×10^{-4}	2.00 (1.68, 2.37)<br>2.5×10^{-15}	1.19 (1.12, 1.27)<br>5.4×10^{-8}
**SLE**	1.03 (0.87, 1.22)<br>*0.73*	1.05 (0.93, 1.19)<br>*0.45*	1.37 (0.70, 2.69)<br>*0.36*	1.04 (0.88, 1.23)<br>*0.64*	1.37 (1.22, 1.54)<br>2.0×10^{-7}	1.34 (1.17, 1.54)<br>3.9×10^{-5}	1.12 (0.99, 1.27)<br>*0.072*	1.13 (0.99, 1.29)<br>*0.074*	2.35 (1.63, 3.39)<br>4.7×10^{-6}	2.00 (1.75, 2.29)<br>9.4×10^{-24}
**T1D**	1.03 (0.68, 1.57)<br>*0.89*	1.05 (0.95, 1.16)<br>*0.35*	1.49 (0.94, 2.37)<br>*0.091*	1.08 (0.96, 1.21)<br>*0.19*	1.07 (0.98, 1.16)<br>*0.11*	1.21 (1.10, 1.33)<br>1.4×10^{-4}	2.00 (1.83, 2.19)<br>6.1×10^{-51}	1.16 (1.05, 1.28)<br>3.0×10^{-3}	1.85 (1.43, 2.39)<br>2.9×10^{-6}	1.10 (1.00, 1.22)<br>0.045
**UC**	2.00 (1.87, 2.14)<br>1.2×10^{-93}	1.51 (1.42, 1.60))<br>4.1×10^{-40}	1.58 (1.17, 2.14)<br>3.0×10^{-3}	1.03 (0.96, 1.10)<br>*0.38*	1.10 (1.04, 1.16)<br>3.1×10^{-4}	1.09 (1.03, 1.16)<br>5.1×10^{-3}	1.06 (1.00, 1.13)<br>*0.061*	1.04 (0.98, 1.10)<br>*0.18*	1.05 (0.90, 1.22)<br>*0.53*	1.00 (0.93, 1.06)<br>*0.89*
**Asthma**	1.02 (0.96, 1.09)<br>*0.54*	1.01 (0.98, 1.04)<br>*0.54*	1.19 (0.94, 1.50)<br>*0.14*	0.97 (0.92, 1.02)<br>*0.24*	0.98 (0.94, 1.02)<br>*0.33*	1.02 (0.97, 1.07)<br>*0.41*	0.98 (0.94, 1.02)<br>*0.30*	0.98 (0.94, 1.02)<br>*0.32*	0.97 (0.87, 1.08)<br>*0.59*	0.99 (0.94, 1.03)<br>*0.56*

Supplementary Table 9: Overview of training studies covering the whole genome and used in polygenic risk score analysis.

Phenotype	# Cases	# Controls	Reference
Psoriasis	1,359	1,400	[40]
Multiple Sclerosis	6,281	5,330	Unpublished
Primary Biliary Cirrhosis	2,764	10,475	[41]
Rheumatoid arthritis	14,361	43,923	[16], European subset
Systemic Lupus Erythematosus	4,036	6,959	[18]
Crohn's disease	5,956	14,927	[39]
Ulcerative colitis	6,968	20,464	[39]

Supplementary Table 10: Summary of most predictive thresholds when PRS from training sets covering the whole genome were tested against their corresponding phenotype in an independent target set. Nagelkerke's pseudo R^2 is reported as a measure of the variance explained.

Training phenotype	Pt	R^2 (%)	P
Psoriasis	1×10^{-6}	0.42	3.3×10^{-24}
Multiple Sclerosis	0.5	0.57	5.9×10^{-8}
Primary Biliary Cirrhosis	1×10^{-4}	2.2	3.9×10^{-9}
Rheumatoid arthritis	1×10^{-5}	0.76	2.8×10^{-18}
Systemic Lupus Erythematosus	0.2	5.7	7.8×10^{-37}
Crohn's disease	1×10^{-5}	1.9	7.2×10^{-17}
Ulcerative colitis	1×10^{-6}	2.4	5.1×10^{-58}

Supplementary Table 11: Number of variants making up each polygenic risk score calculated from studies covering the whole genome. The most predictive threshold is shown in bold.

Phenotype	1E-6	1E-5	1E-4	1E-3	1E-2	0.1	0.2	0.3	0.4	0.5
Multiple Sclerosis	23	49	157	834	5,837	38,414	64,534	85,467	102,820	**117,252**
Rheumatoid arthritis	96	**144**	308	1,085	6,307	39,080	64,063	85,174	102,926	117,659
Crohn's disease	119	**196**	440	1,730	8,746	44,777	70,863	90,859	106,660	119,676
Ulcerative colitis	**72**	132	370	1,639	8,907	46,892	73,913	94,448	111,113	124,521
Psoriasis	**4**	11	50	316	2,535	17,393	29,562	39,734	48,310	55,593
Systemic Lupus Erythematosus	88	164	497	2,333	12,773	64,700	**100,451**	127,632	149,251	166,965
Primary Biliary Cirrhosis	44	77	**179**	682	3,701	21,697	35,745	47,419	57,152	65,558

Supplementary Table 12: Results of polygenic risk score analysis for training studies covering the whole genome. Risk ratios and 95% confidence intervals for each disease pair with P-values in italic below. Diseases for which we calculated polygenic risk score are listed horizontally while diseases for which an Icelandic cohort was available are listed vertically.

Disease	MS	CD	UC	RA	PSO	SLE	PBC
**AS**	1.26 (0.92, 1.73)	1.32 (1.05, 1.52)	1.24 (1.05, 1.47)	1.03 (0.72, 1.47)	0.82 (0.51, 1.33)	1.04 (0.94, 1.16)	1.03 (0.89, 1.21)
	0.15	1.5×10^{-3}	*0.013*	*0.87*	*0.42*	*0.42*	*0.66*
**AITD**	0.93 (0.79, 1.09)	0.95 (0.86, 1.05)	0.97 (0.90, 1.05)	1.79 (1.54, 2.08)	1.00 (0.99, 1.01)	1.05 (0.99, 1.11)	1.08 (1.00, 1.16)
	0.37	*0.23*	*0.43*	6.4×10^{-14}	*0.98*	*0.11*	*0.062*
**CD**	0.87 (0.64, 1.18)	2.00 (1.68, 2.39)	1.61 (1.36, 1.90)	1.28 (0.96, 1.71)	1.08 (0.66, 1.77)	1.05 (0.95, 1.17)	1.00 (0.87, 1.16)
	0.37	7.2×10^{-17}	2.1×10^{-8}	*0.096*	*0.76*	*0.31*	*0.95*
**MS**	2.00 (1.56, 2.56)	1.01 (0.98, 1.02)	1.09 (0.95, 1.25)	1.09 (0.86, 1.38)	1.40 (0.96, 2.05)	1.02 (0.94, 1.11)	1.28 (1.14, 1.44)
	3.1×10^{-8}	*0.94*	*0.21*	*0.47*	*0.082*	*0.62*	4.6×10^{-5}
**PBC**	1.63 (1.01, 2.63)	0.95 (0.86, 1.49)	1.16 (0.90, 1.50)	2.31 (1.47, 3.62)	1.06 (0.53, 2.13)	1.20 (1.02, 1.41)	2.00 (1.59, 2.52)
	0.045	*0.69*	*0.26*	2.6×10^{-4}	*0.87*	*0.027*	3.9×10^{-9}
**PSO**	1.09 (1.00, 1.19)	1.12 (1.08, 1.19)	1.08 (1.03, 1.13)	0.97 (0.89, 1.06)	2.00 (1.75, 2.29)	0.99 (0.96, 1.02)	1.12 (1.07, 1.17)
	0.044	1.2×10^{-6}	1.5×10^{-3}	*0.50*	3.3×10^{-24}	0.53	2.7×10^{-7}
**RA**	1.00 (0.98, 1.02)	1.02 (1.00, 1.00)	1.00 (0.86, 1.17)	2.00 (1.72, 2.32)	0.87 (0.68, 1.11)	1.11 (1.06, 1.18)	1.10 (1.02, 1.19)
	0.99	*0.71*	*0.99*	1.6×10^{-19}	*0.27*	6.8×10^{-5}	*0.011*
**SLE**	0.96 (0.71, 1.30)	1.15 (0.89, 1.31)	0.95 (0.79, 1.14)	2.38 (1.73, 3.27)	1.52 (0.90, 2.58)	2.00 (1.80, 2.23)	1.50 (1.28, 1.77)
	0.79	*0.14*	*0.58*	8.1×10^{-8}	*0.12*	7.8×10^{-37}	1.6×10^{-6}
**T1D**	0.96 (0.77, 1.20)	1.03 (0.90, 1.22)	1.00 (0.95, 1.05)	2.10 (1.68, 2.63)	0.90 (0.62, 1.31)	1.10 (1.01, 1.19)	1.20 (1.07, 1.34)
	0.72	*0.63*	*0.97*	7.5×10^{-11}	*0.58*	*0.028*	*0.0024*
**UC**	1.03 (0.88, 1.20)	1.51 (1.37, 1.64)	2.00 (1.84, 2.18)	1.13 (0.98, 1.31)	1.13 (0.90, 1.42)	0.99 (0.94, 1.05)	1.02 (0.95, 1.10)
	0.71	3.6×10^{-22}	5.1×10^{-58}	*0.10*	*0.30*	*0.77*	*0.52*
**Asthma**	1.07 (0.96, 1.20)	1.00 (0.90, 1.04)	0.99 (0.93, 1.05)	0.94 (0.85, 1.04)	1.00 (0.98, 1.02)	1.02 (0.99, 1.07)	0.96 (0.90, 1.01)
	0.24	*0.94*	*0.75*	*0.25*	*0.99*	*0.21*	*0.13*

Supplementary Table 13: Association statistics for SNPs identified through the proxy-phenotype method in each study cohort individually. Effect allele (and minor allele in Iceland) is enclosed in brackets.

	IMSGC meta-analysis		Swedish cohort		Icelandic cohort		Norwegian cohort	
**rsID**	**P**	**OR**	**P**	**OR**	**P**	**OR**	**P**	**OR**
rs72678531 (G)	9.3×10^{-4}	1.07 (1.03, 1.12)	3.6×10^{-4}	1.16 (1.07, 1.26)	0.33	1.06 (0.94, 1.20)	0.80	1.02 (0.90, 1.14)
rs17674224 (C)	0.036	1.03 (1.00, 1.07)	0.078	1.05 (0.99, 1.11)	0.074	1.09 (0.99, 1.21)	0.0014	1.16 (1.06, 1.27)
rs35188261 (A)	0.0017	1.08 (1.03, 1.14)	0.0099	1.11 (1.03, 1.21)	0.99	1.00 (0.82, 1.23)	0.36	1.08 (0.92, 1.26)
rs12871645 (A)[a]	6.3×10^{-4}	0.88 (0.82, 0.95)	0.99	0.92 (0.80, 1.06)	0.032	0.75 (0.58, 0.98)	0.97	0.95 (0.75, 1.20)
rs2271293 (A)	0.015	1.06 (1.01, 1.11)	0.083	1.06 (0.99, 1.13)	0.0063	1.24 (1.06, 1.44)	0.0061	1.18 (1.05, 1.33)
rs35018800 (A)	0.0013	0.72 (0.59, 0.88)	0.060	0.68 (0.45, 1.02)	0.0011	0.36 (0.19, 0.66)	0.95	0.97 (0.35, 2.69)
rs2073167 (G)	2.2×10^{-5}	0.93 (0.90, 0.96)	0.066	0.95 (0.90, 1.00)	0.78	1.01 (0.92, 1.12)	0.21	0.95 (0.88, 1.03)

[a]Conditioned on rs806321.

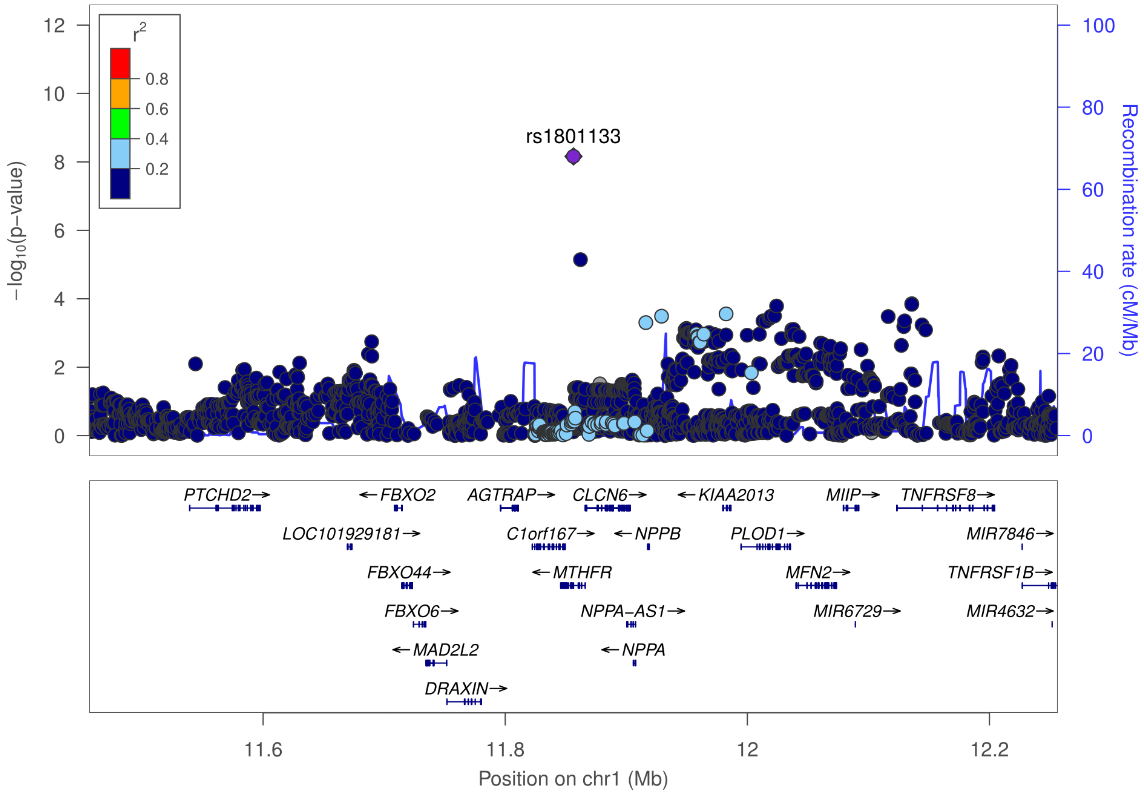

Supplementary Figure 1: Locus plot for rs1801133

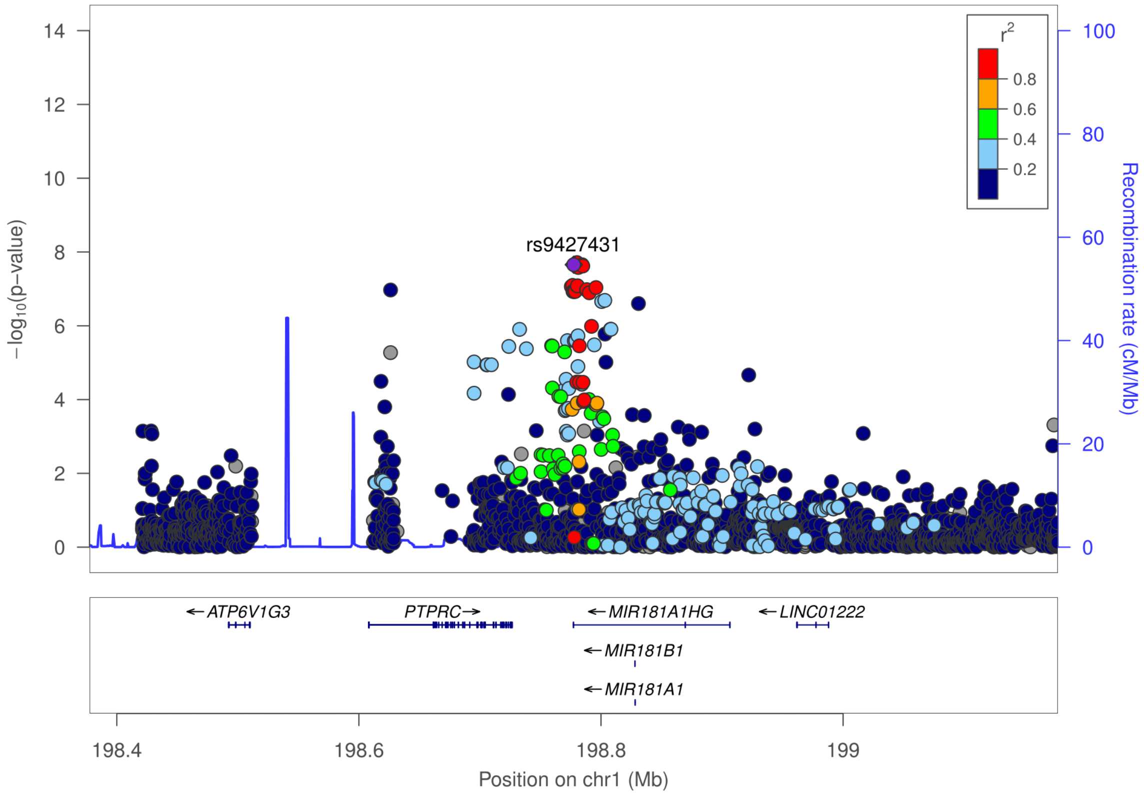

Supplementary Figure 2: Locus plot for rs9427431

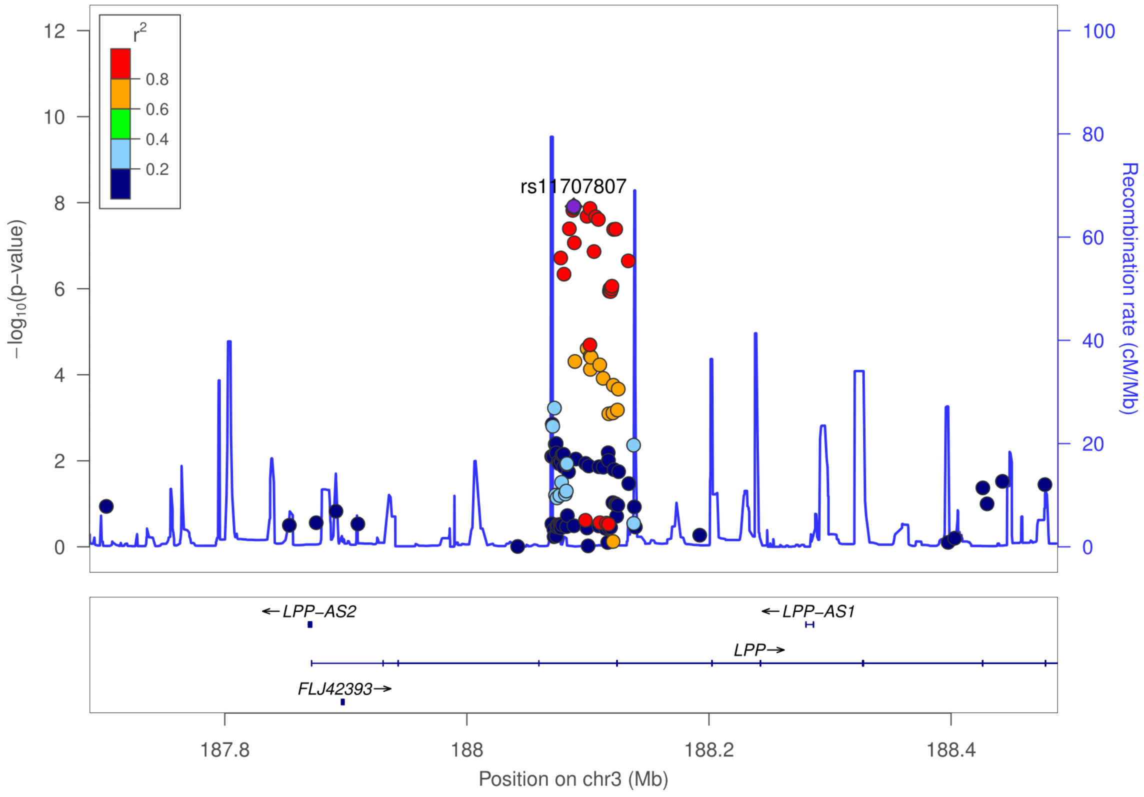

Supplementary Figure 3: Locus plot for rs11707807.

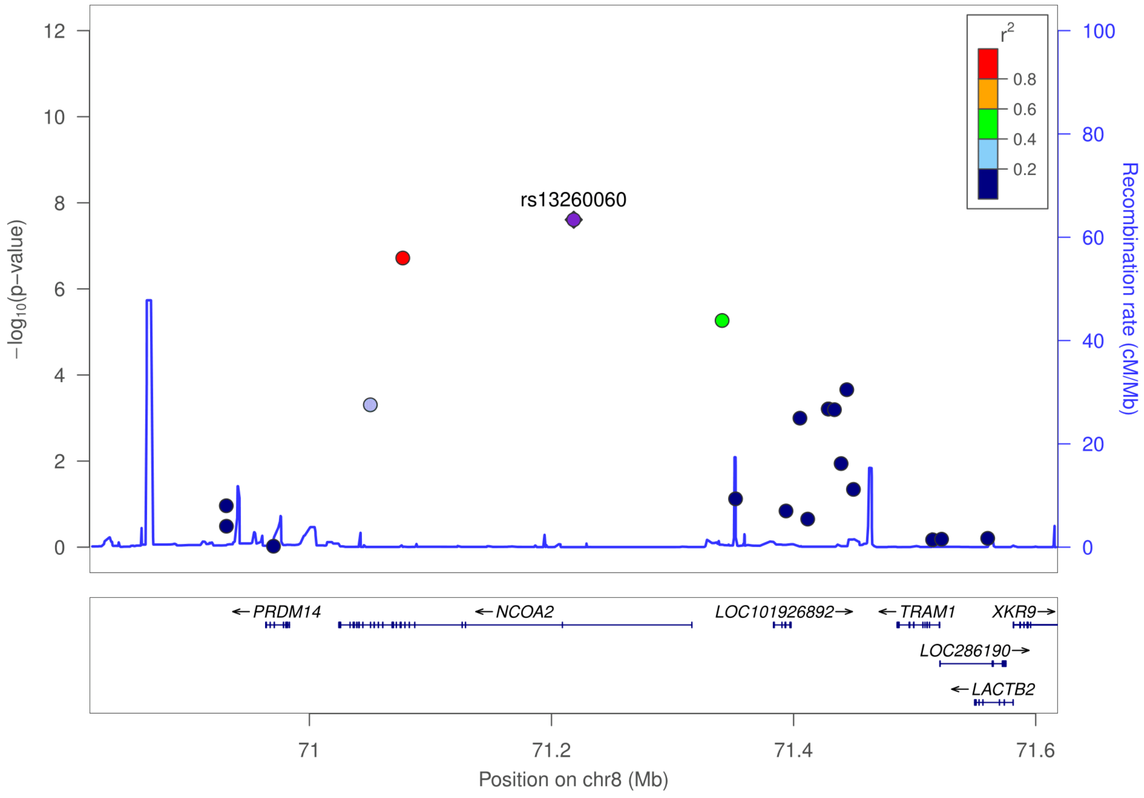

Supplementary Figure 4: Locus plot for rs13260060.

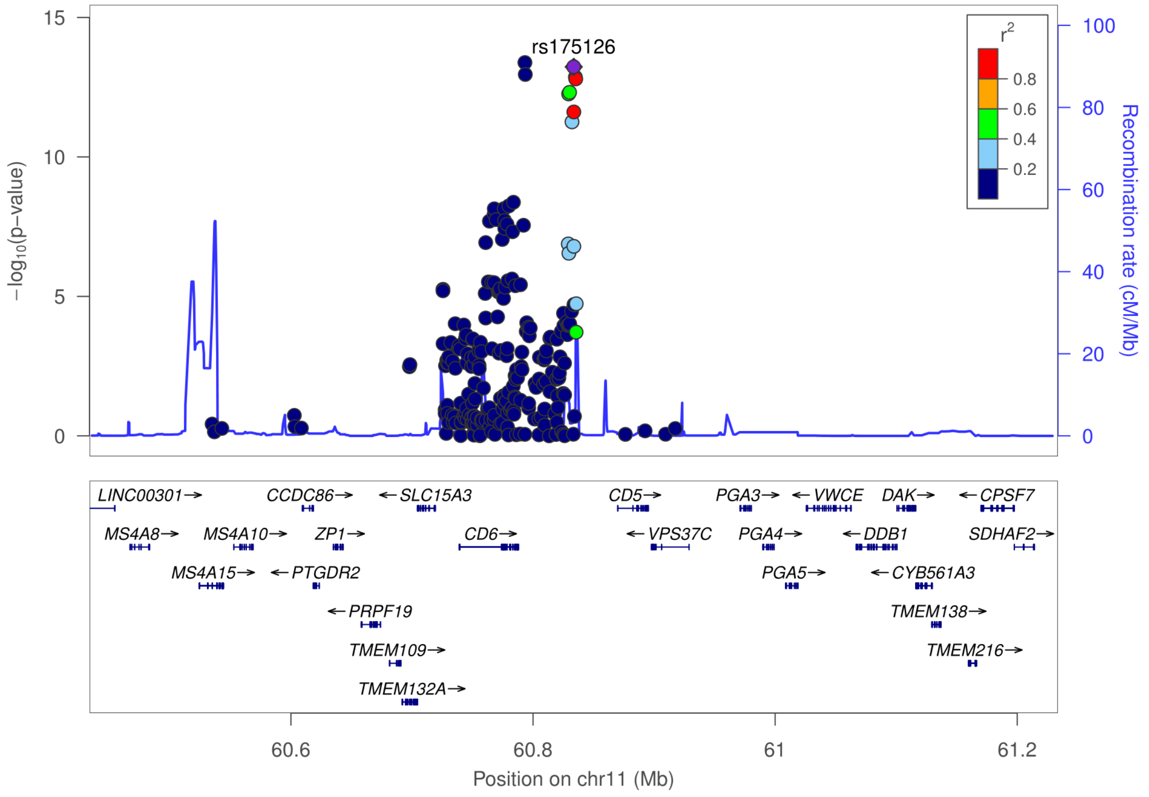

Supplementary Figure 5: Locus plot for rs175126.

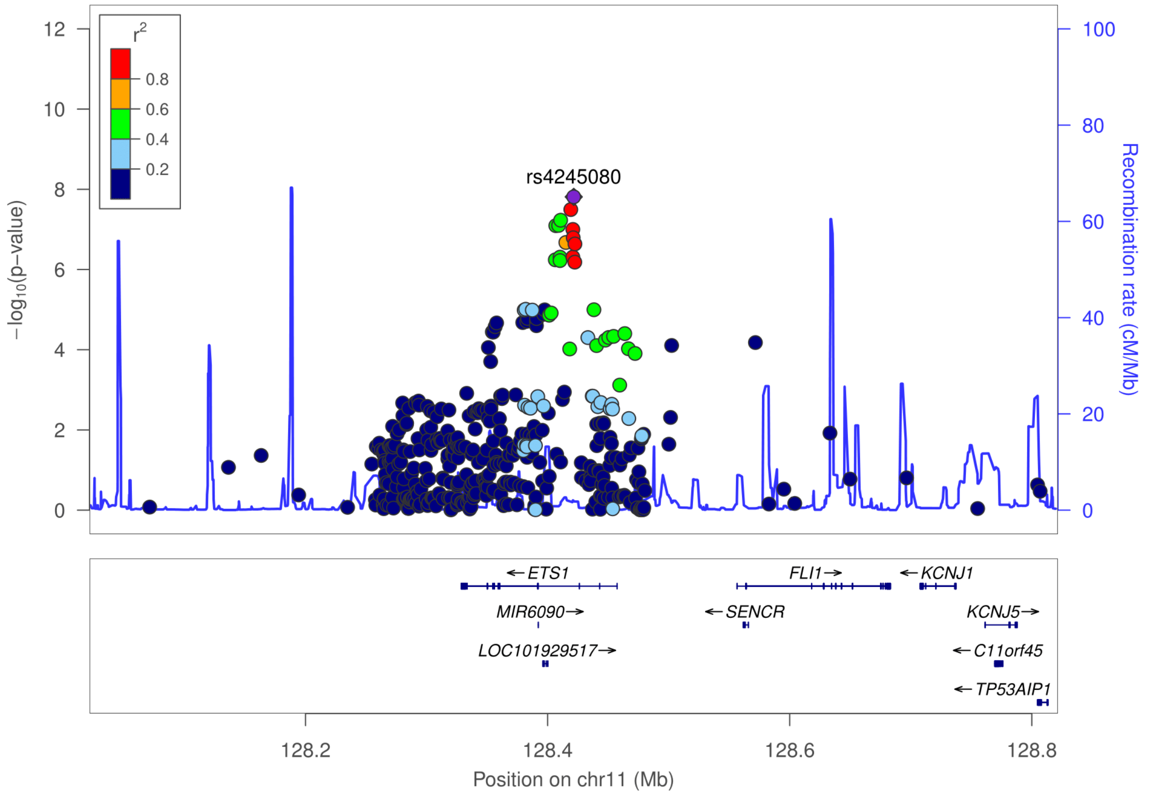

Supplementary Figure 6: Locus plot for rs4245080.

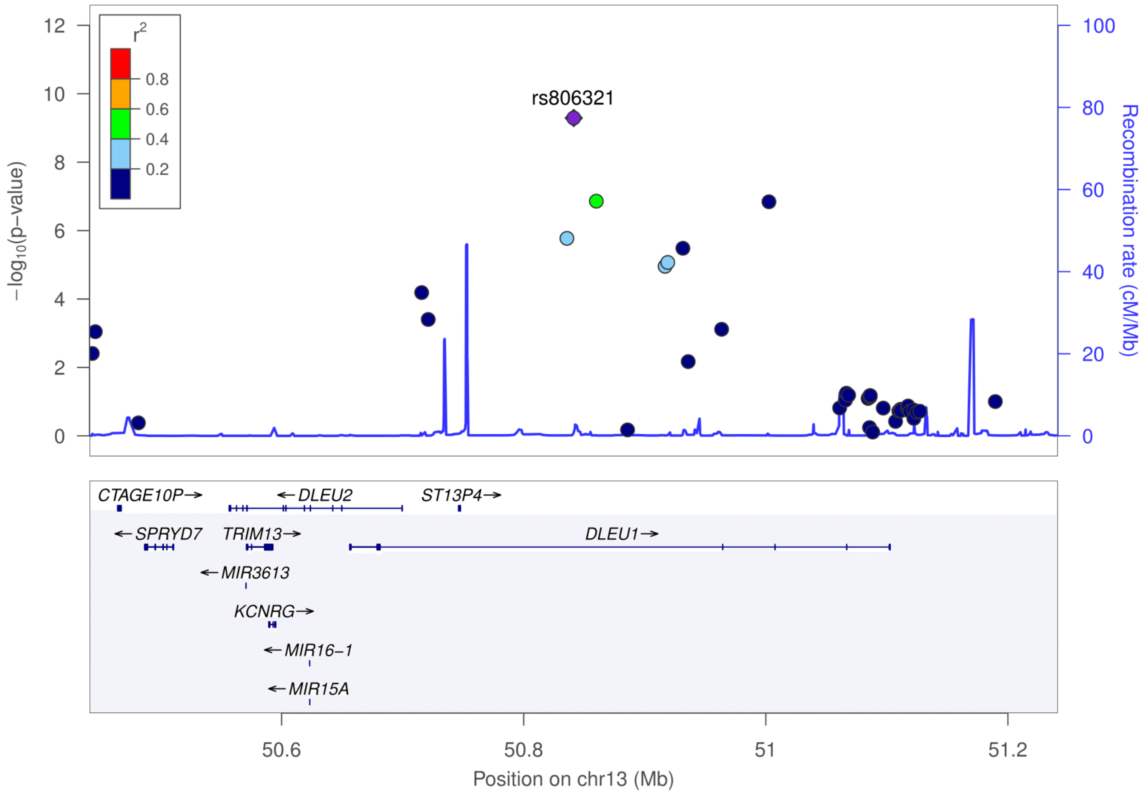

Supplementary Figure 7: Locus plot for rs806321.

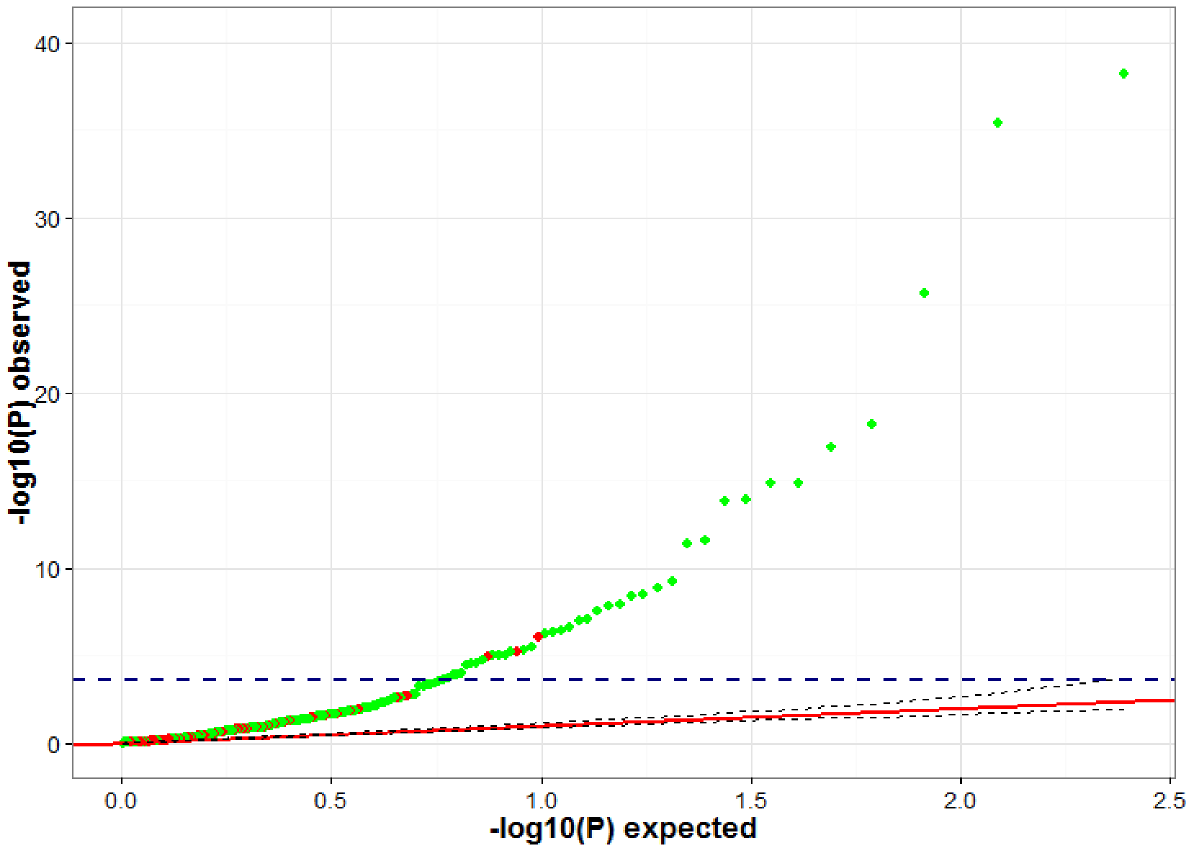

Supplementary Figure 8: Q-Q plot for variants from the most predictive polygenic risk score for Primary biliary cirrhosis associating with Multiple sclerosis. Green indicates concordance of effect between the two studies while red indicates discordance. Dashed blue line indicates the significance threshold of 2.0×10^{-4}.

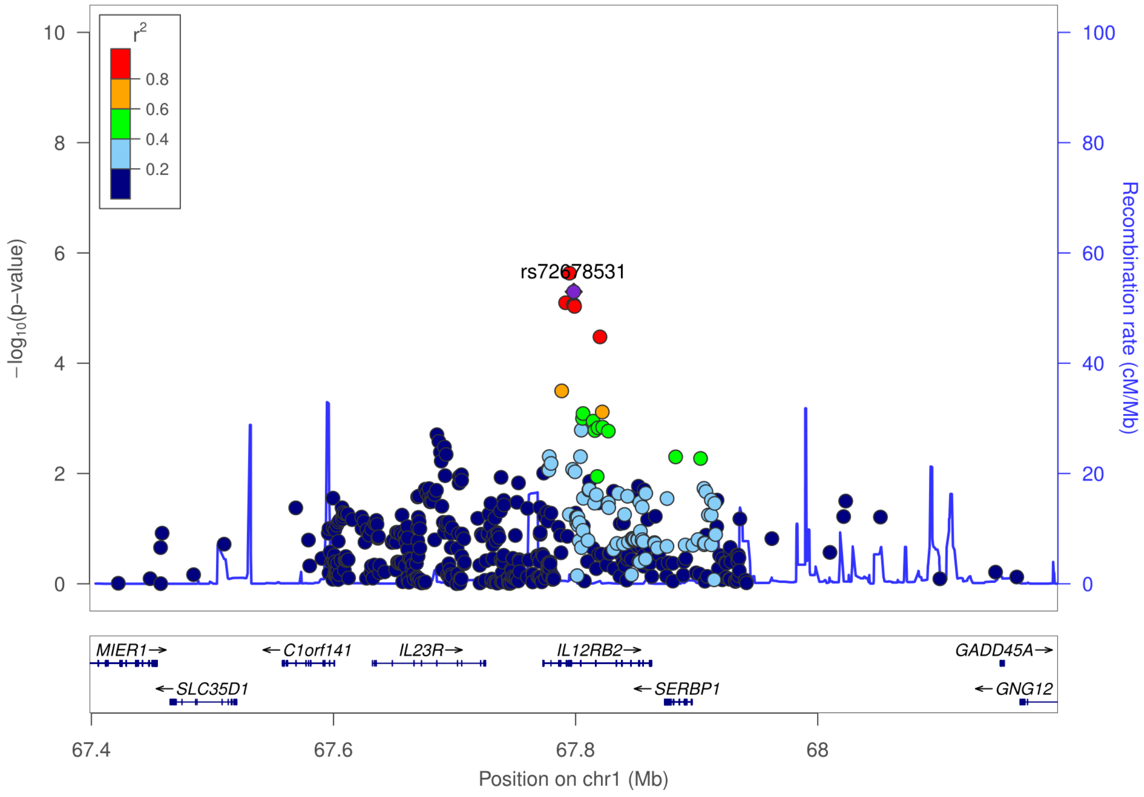

Supplementary Figure 9: Locus plot for rs72678531.

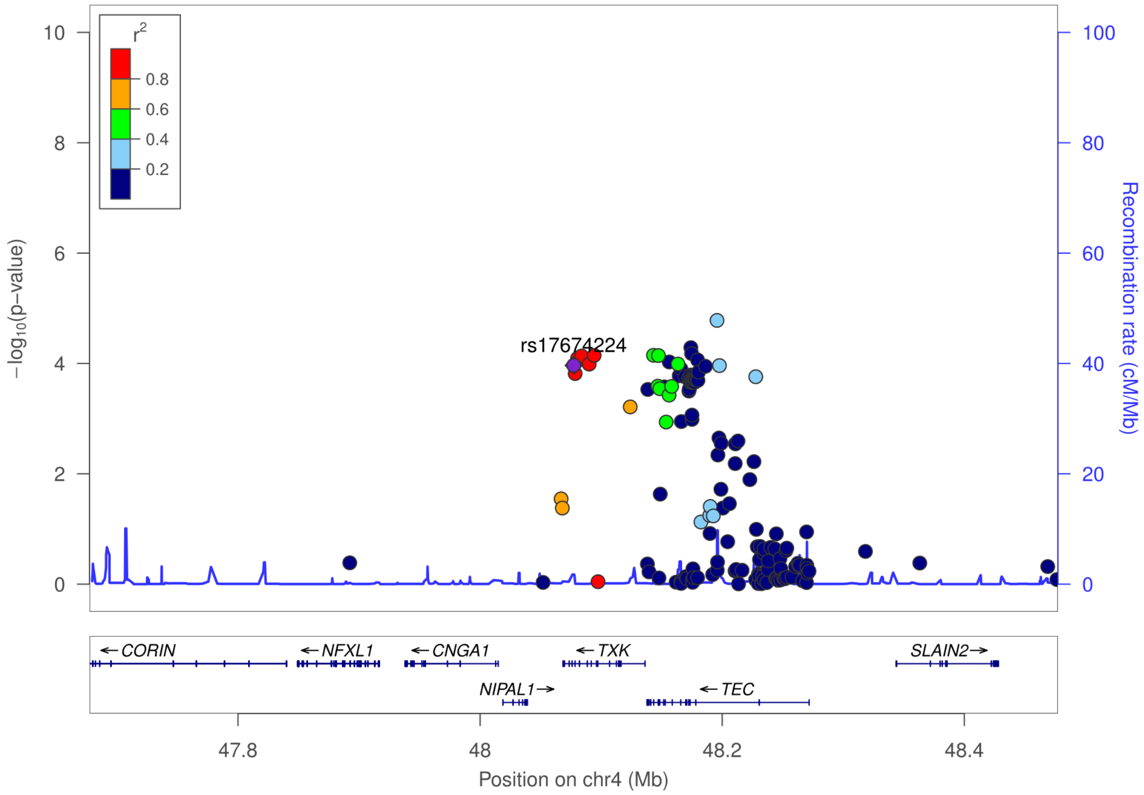

Supplementary Figure 10: Locus plot for rs17674224.

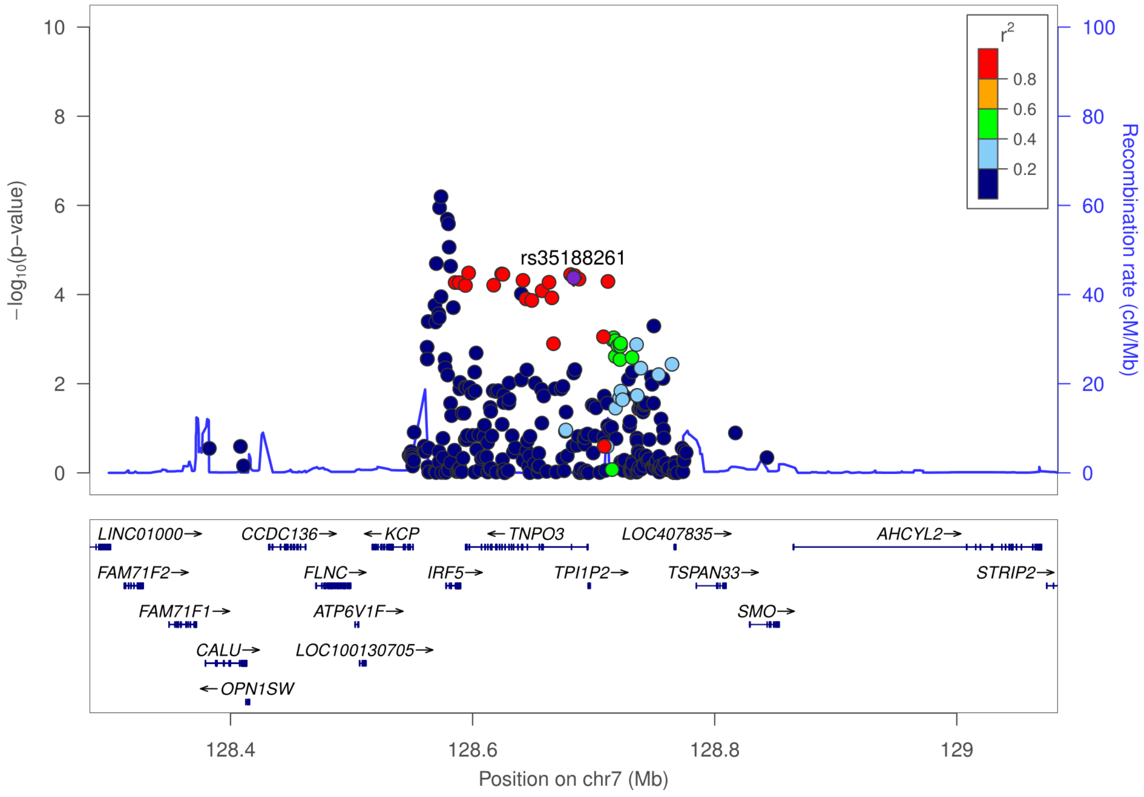

Supplementary Figure 11: Locus plot for rs35188261.

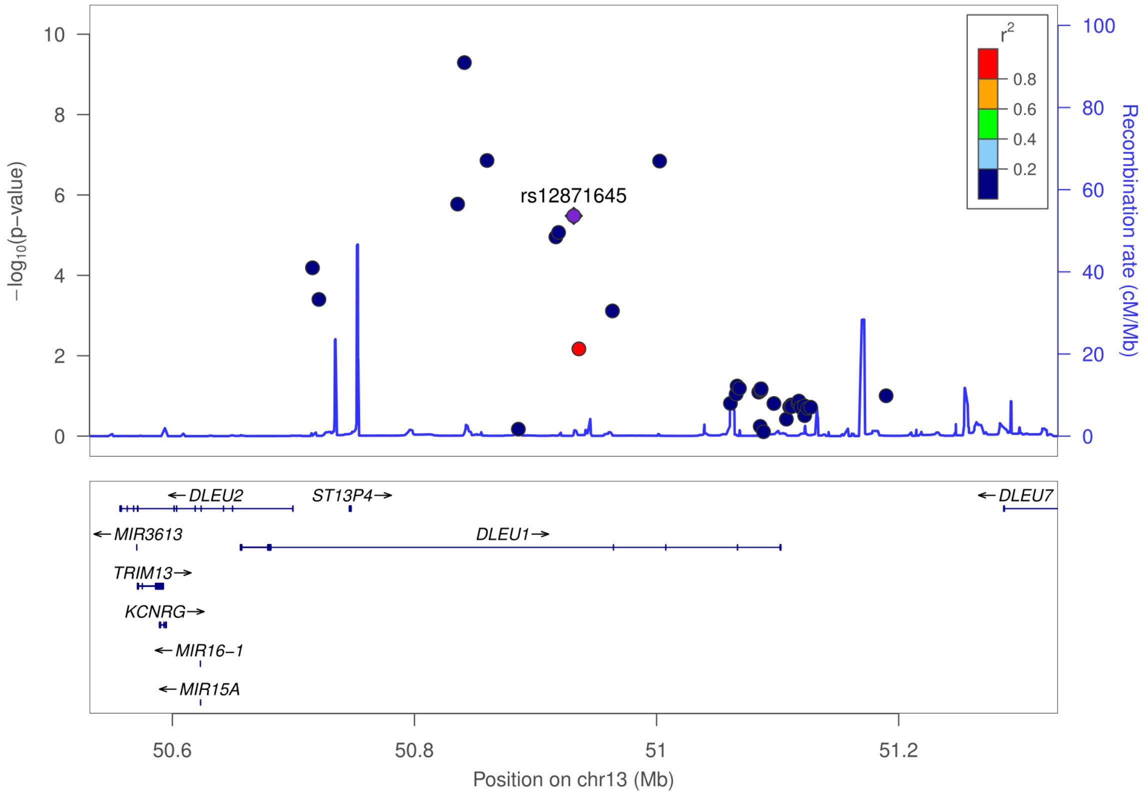

Supplementary Figure 12: Locus plot for rs12871645.

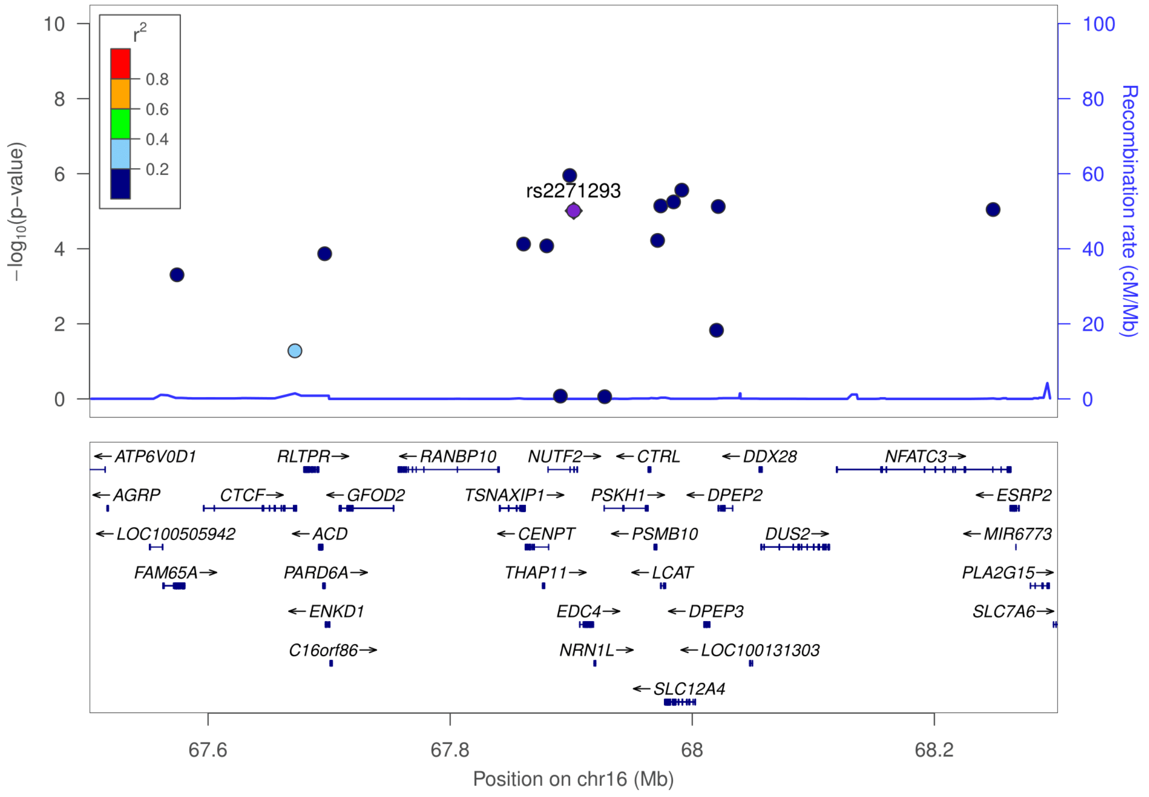

Supplementary Figure 13: Locus plot for rs2271293.

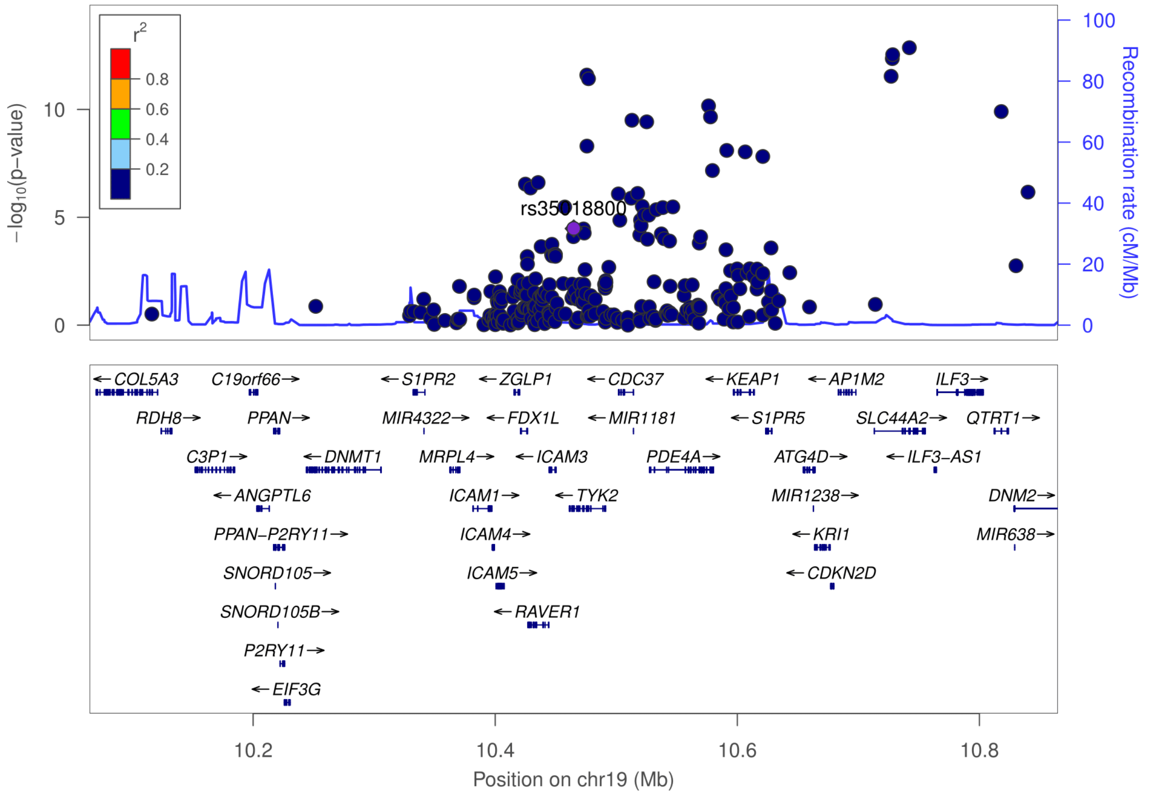

Supplementary Figure 14: Locus plot for rs35018800.

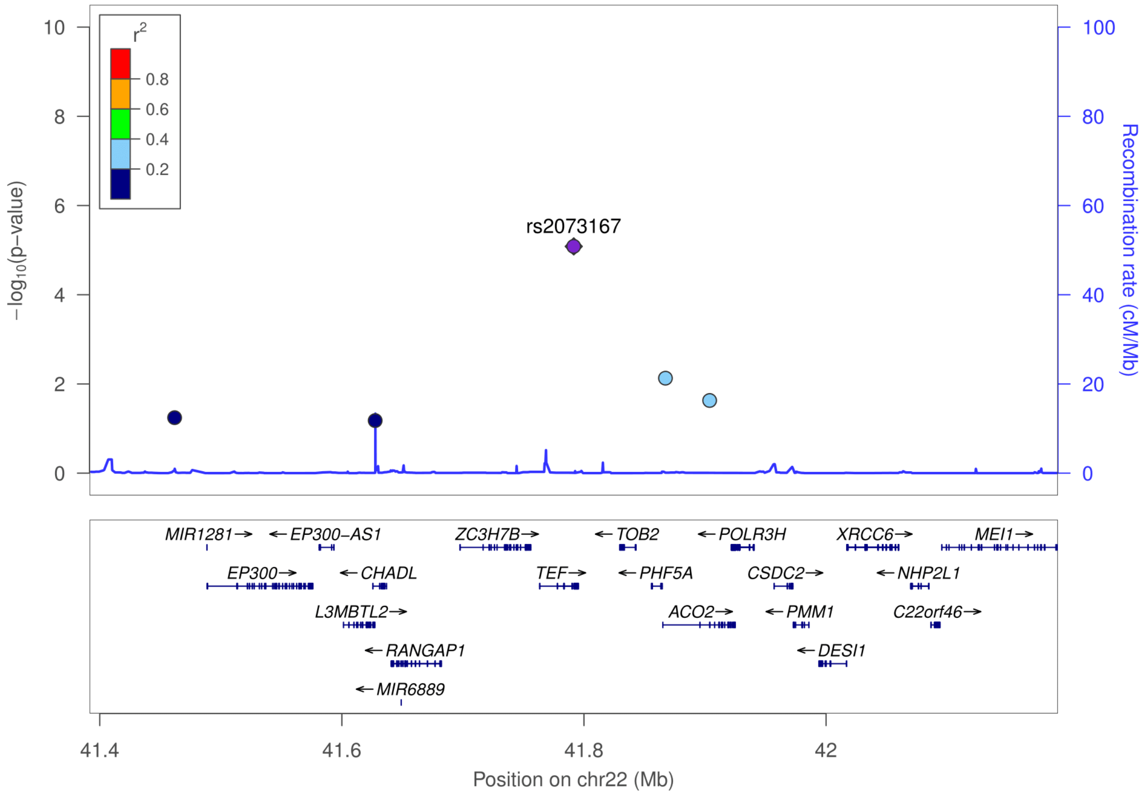

Supplementary Figure 15: Locus plot for rs2073167.

Supplementary references

1. Klerk, M. *et al.* MTHFR 677C-->T polymorphism and risk of coronary heart disease: a meta-analysis. *JAMA* **288,** 2023–31 (2010).

2. Mao, R. *et al.* Association study between methylenetetrahydrofolate reductase gene polymorphisms and Graves' disease. *Cell Biochem. Funct.* **28,** 585–90 (2010).

3. Pingel, J. T. *et al.* Evidence that the leukocyte-common antigen is required for antigen-induced T lymphocyte proliferation. *Cell* **58,** 1055–65 (1989).

4. Hemmer, B. *et al.* A point mutation in PTPRC is associated with the development of multiple sclerosis. *Nat. Genet.* **26,** 495–499 (2000).

5. Barcellos, L. F. *et al.* PTPRC (CD45) is not associated with the development of multiple sclerosis in U.S. patients. *Nat. Genet.* **29,** 23–4 (2001).

6. Cocco, E. *et al.* PTPRC (CD45) C77G mutation does not contribute to multiple sclerosis susceptibility in Sardinian patients. *J. Neurol.* **251,** 1085–8 (2004).

7. Trynka, G. *et al.* Dense genotyping identifies and localizes multiple common and rare variant association signals in celiac disease. *Nat. Genet.* **43,** 1193–201 (2011).

8. Cooper, J. D. *et al.* Seven newly identified loci for autoimmune thyroid disease. *Hum. Mol. Genet.* **21,** 5202–8 (2012).

9. Jin, Y. *et al.* Genome-wide association analyses identify 13 new susceptibility loci for generalized vitiligo. *Nat. Genet.* **44,** 676–80 (2012).

10. Hong, H., Kohli, K., Garabedian, M. J. & Stallcup, M. R. GRIP1, a transcriptional coactivator for the AF-2 transactivation domain of steroid, thyroid, retinoid, and vitamin D receptors. *Mol. Cell. Biol.* **17,** 2735–44 (1997).

11. Bledsoe, R. K. *et al.* Crystal Structure of the Glucocorticoid Receptor Ligand Binding Domain Reveals a Novel Mode of Receptor Dimerization and Coactivator Recognition. *Cell* **110,** 93–105 (2002).

12. Takeyama, K. *et al.* Selective interaction of vitamin D receptor with transcriptional coactivators by a vitamin D analog. *Mol. Cell. Biol.* **19,** 1049–55 (1999).

13. Heintzman, N. D. *et al.* Distinct and predictive chromatin signatures of transcriptional promoters and enhancers in the human genome. *Nat. Genet.* **39,** 311–318 (2007).

14. Zamisch, M. *et al.* The transcription factor Ets1 is important for CD4 repression and Runx3 up-regulation during CD8 T cell differentiation in the thymus. *J. Exp. Med.* **206,** 2685–99 (2009).

15. Moisan, J., Grenningloh, R., Bettelli, E., Oukka, M. & Ho, I.-C. Ets-1 is a negative regulator of Th17 differentiation. *J. Exp. Med.* **204,** 2825–35 (2007).

16. Okada, Y. *et al.* Genetics of rheumatoid arthritis contributes to biology and drug discovery. *Nature* **506,** 376–81 (2014).

17. Tsoi, L. C. *et al.* Identification of 15 new psoriasis susceptibility loci highlights the role of innate immunity. *Nat. Genet.* **44,** 1341–1348 (2012).

18. Bentham, J. *et al.* Genetic association analyses implicate aberrant regulation of innate and adaptive immunity genes in the pathogenesis of systemic lupus erythematosus. *Nat. Genet.* **47,** 1457–1464 (2015).

19. Creyghton, M. P. *et al.* Histone H3K27ac separates active from poised enhancers and predicts developmental state. *Proc. Natl. Acad. Sci. U. S. A.* **107,** 21931–6 (2010).

20. Hsieh, C. S. *et al.* Development of TH1 CD4+ T cells through IL-12 produced by Listeria-induced macrophages. *Science (80-.).* **260,** 547–9 (1993).

21. Liu, J. Z. *et al.* Dense fine-mapping study identifies new susceptibility loci for primary biliary cirrhosis. *Nat. Genet.* **44,** 1137–1141 (2012).

22. Mizuki, N. *et al.* Genome-wide association studies identify IL23R-IL12RB2 and IL10 as Behçet's disease susceptibility loci. *Nat. Genet.* **42,** 703–706 (2010).

23. Bossini-Castillo, L. *et al.* A GWAS follow-up study reveals the association of the IL12RB2 gene with systemic sclerosis in Caucasian populations. *Hum. Mol. Genet.* **21,** 926–33 (2012).

24. Jostins, L. *et al.* Host–microbe interactions have shaped the genetic architecture of inflammatory bowel disease. *Nature* **491,** 119–124 (2012).

25. Raelson, J. V *et al.* Genome-wide association study for Crohn's disease in the Quebec Founder Population identifies multiple validated disease loci. *Proc. Natl. Acad. Sci. U. S. A.* **104,** 14747–52 (2007).

26. Zhang, G.-X. *et al.* Induction of experimental autoimmune encephalomyelitis in IL-12 receptor-beta 2-deficient mice: IL-12 responsiveness is not required in the pathogenesis of inflammatory demyelination in the central nervous system. *J. Immunol.* **170,** 2153–60 (2003).

27. Nagafuchi, H. *et al.* Excessive expression of Txk, a member of the Tec family of tyrosine kinases, contributes to excessive Th1 cytokine production by T lymphocytes in patients with Behcet's disease. *Clin. Exp. Immunol.* **139,** 363–70 (2005).

28. Armstrong, D. L. *et al.* GWAS identifies novel SLE susceptibility genes and explains the association of the HLA region. *Genes Immun.* **15,** 347–54 (2014).

29. Eyre, S. *et al.* High-density genetic mapping identifies new susceptibility loci for rheumatoid arthritis. *Nat. Genet.* **44,** 1336–1340 (2012).

30. Miceli-Richard, C. *et al.* The CGGGG insertion/deletion polymorphism of the IRF5 promoter is a strong risk factor for primary Sjögren's syndrome. *Arthritis Rheum.* **60,** 1991–7 (2009).

31. Dieudé, P. *et al.* Association between the IRF5 rs2004640 functional polymorphism and systemic sclerosis: a new perspective for pulmonary fibrosis. *Arthritis Rheum.* **60,** 225–33 (2009).

32. International Multiple Sclerosis Genetics Consortium (IMSGC) *et al.* Analysis of immune-related loci identifies 48 new susceptibility variants for multiple sclerosis. *Nat. Genet.* **45,** 1353–60 (2013).

33. Ivanov, P. & Anderson, P. Post-transcriptional regulatory networks in immunity. *Immunol. Rev.* **253,** 253–72 (2013).

34. Aulchenko, Y. S. *et al.* Loci influencing lipid levels and coronary heart disease risk in 16 European population cohorts. *Nat. Genet.* **41,** 47–55 (2009).

35. Hinks, A. *et al.* Dense genotyping of immune-related disease regions identifies 14 new susceptibility loci for juvenile idiopathic arthritis. *Nat. Genet.* **45,** 664–669 (2013).

36. Sigurdsson, S. *et al. Polymorphisms in the Tyrosine Kinase 2 and Interferon Regulatory Factor 5 Genes Are Associated with Systemic Lupus Erythematosus*. *The American Journal of Human Genetics* **76,** (2005).

37. Onengut-Gumuscu, S. *et al.* Fine mapping of type 1 diabetes susceptibility loci and evidence for colocalization of causal variants with lymphoid gene enhancers. *Nat. Genet.* **47,** 381–386 (2015).

38. Drolet, D. W. *et al.* TEF, a transcription factor expressed specifically in the anterior pituitary during embryogenesis, defines a new class of leucine zipper proteins. *Genes Dev.* **5,** 1739–53 (1991).

39. Liu, J. Z. *et al.* Association analyses identify 38 susceptibility loci for inflammatory bowel disease and highlight shared genetic risk across populations. *Nat. Genet.* **47,** 979–986 (2015).

40. Feng, B.-J. *et al.* Multiple Loci within the Major Histocompatibility Complex Confer Risk of Psoriasis. *PLoS Genet.* **5,** e1000606 (2009).

41. Cordell, H. J. *et al.* International genome-wide meta-analysis identifies new primary biliary cirrhosis risk loci and targetable pathogenic pathways. *Nat. Commun.* **6,** 8019 (2015).
